# Iulomorphid millipedes (Diplopoda, Spirostreptida, Iulomorphidae) of Tasmania, Australia

**DOI:** 10.3897/zookeys.652.12035

**Published:** 2017-02-06

**Authors:** Robert Mesibov

**Affiliations:** 1 West Ulverstone, Tasmania 7315, Australia

**Keywords:** Diplopoda, Spirostreptida, Iulomorphidae, Tasmania, Australia

## Abstract

Tasmanian Iulomorphidae are here assigned to the genera *Amastigogonus* Brölemann, 1913, *Atelomastix* Attems, 1911 and *Equestrigonus* **gen. n.** Descriptions or redescriptions are given for *Amastigogonus danpicola* **sp. n.**, *Amastigogonus elephas* **sp. n.**, *Amastigogonus fossuliger* Verhoeff, 1944, *Amastigogonus hardyi* (Chamberlin, 1920), *Amastigogonus hellyeri* **sp. n.**, *Amastigogonus michaelsae* **sp. n.**, *Amastigogonus orientalis* **sp. n.**, *Amastigogonus peninsulensis* **sp. n.**, *Amastigogonus tasmanianus* Brölemann, 1913 (type species of *Amastigogonus*), *Amastigogonus verreauxii* (Gervais, 1847), *Atelomastix bonhami* **sp. n.**, *Atelomastix smithi* **sp. n.** and *Equestrigonus tasmaniensis* **gen. n., sp. n.** The synonymy of *Amastigogonus nichollsii* Verhoeff, 1944 with *Amastigogonus hardyi* is accepted, and lectotypes are designated for *Amastigogonus nichollsii* and *Amastigogonus tasmanianus*.

## Introduction

**Figure 1.**
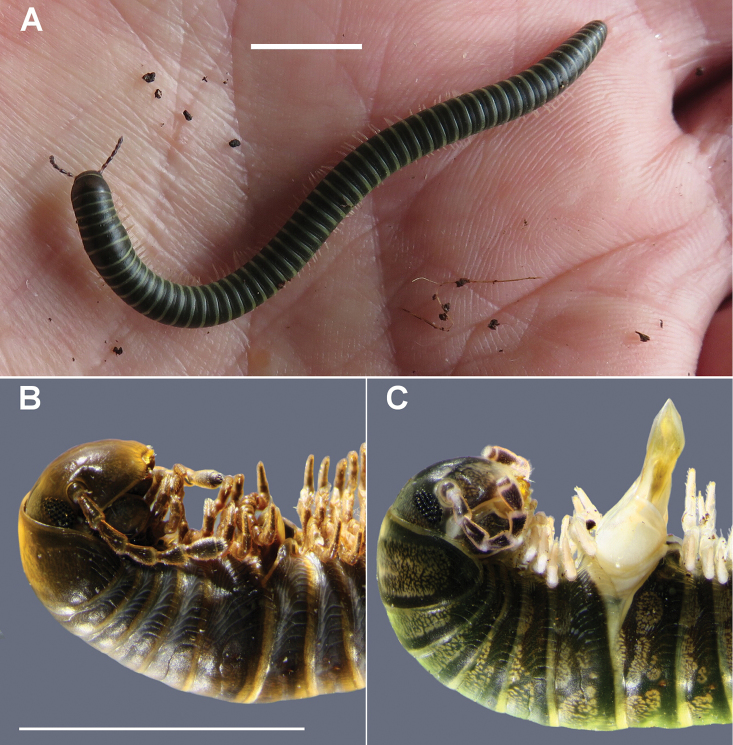
*Amastigogonus fossuliger* Verhoeff, 1944. **A** Living male on author’s palm, later preserved in QVM 23:54468 **B** Long-preserved male with retracted gonopods, ex QVM 23:54290 **C** Male with gonopods everted after freeze-killing and soaking in water, ex QVM 23:54468. Scale bars: **A** = 10 mm, **B, C** = 5 mm.

In Tasmania, Australia, native species of Spirostreptida are found at all elevations and in most natural habitats, although they are rarely seen in coastal dune scrubs or in grasslands and moorlands. Spirostreptidans can be abundant in native forest and in *Eucalyptus* and *Pinus radiata* plantations. For example, spirostreptidans made up 65% (Mesibov 1993) and 48% (Mesibov 1998) of all millipedes collected in two of the author’s hand-sampling studies in wet eucalypt forest and cool temperate rainforest. Spirostreptida include Tasmania’s longest millipedes (Fig. 1A) and are well-known to many Tasmanian naturalists for the strong smell of their benzoquinone defensive secretions.

Although the spirostreptidan family Cambalidae Bollman, 1893 occurs in Tasmania (Mesibov, in preparation), the most frequently collected spirostreptidans are in Iulomorphidae Verhoeff, 1924 as circumscribed by Korsós and Read (2012). The dominant iulomorphid genus is *Amastigogonus* Brölemann, 1913, which is endemic to Tasmania and has five named species:

1. *Amastigogonus fossuliger* Verhoeff, 1944.
2. *Amastigogonus hardyi* (Chamberlin, 1920). Described as *Euethogonus hardyi*, assigned to *Amastigogonus* by Hoffman (1972).
3. *Amastigogonus nichollsii* Verhoeff, 1944. Synonymised with *Amastigogonus hardyi* by Hoffman (1972).
4. *Amastigogonus tasmanianus* Brölemann, 1913, type species.
5. *Amastigogonus verreauxii* (Gervais, 1847). Described as *Iulus Verreauxii*, assigned to *Amastigogonus* by Mauriès, Golovatch and Hoffman (2001).

In the present study *Amastigogonus fossuliger*, *Amastigogonus hardyi*, *Amastigogonus tasmanianus* and *Amastigogonus verreauxii* are redescribed, lectotypes are designated for *Amastigogonus nichollsii* and *Amastigogonus tasmanianus*, and six new Tasmanian species are added to *Amastigogonus*. I also describe two new Tasmanian species of *Atelomastix* Attems, 1911, a genus previously known only from mainland Australia, and I propose a new genus for a distinctive iulomorphid species which is widespread and locally abundant in the north of Tasmania’s main island.

## Materials and methods

While preparing this paper I supplemented material in the collections of the Queen Victoria Museum and Art Gallery and the Tasmanian Museum and Art Gallery with fresh specimens from selected localities. Iulomorphid millipedes were hand collected in or under woody litter during the day, as well as on tree trunks at night.

In descriptions of individual spirostreptidans I follow Enghoff et al. (1993) in counting trunk rings by excluding the telson and giving podous + apodous ring counts, e.g. “(55+1) rings”, and I give the count ranges I observed rather than count frequencies. In *Atelomastix* species, the three branches of the anterior gonopod are here called sclerites “a”, “b” and “c”, following Attems (1911) and Edward and Harvey (2010). *Amastigogonus* species descriptions only include diagnostically relevant features and the genus description for *Amastigogonus* should be consulted for other details.

Long-preserved Spirostreptida are often deeply stained and made brittle by defensive secretion, and it is difficult to dissect such specimens to examine gonopods without breaking the rings adjoining the gonopod aperture. However, *Amastigogonus* males usually have everted gonopods when first killed by freezing, then submerged in tap water for several hours at room temperature (Fig. 1B, C). I used this procedure to prepare some fresh *Amastigogonus* specimens for examination and description.

Another methodological problem is that the pseudoflagellum on the anterior gonopod of several *Amastigogonus* species is remarkably thin and fragile. In this paper I provide gonopod drawings rather than scanning electron micrographs, as the SEM facility to which I have access does not have a critical-point dryer, and even brief drying can damage or distort an *Amastigogonus* pseudoflagellum.

Dissected gonopods and other body parts were first cleared in 80% lactic acid, then temporarily mounted in 1:1 glycerine:water and imaged using an eyepiece video camera mounted on an Amscope binocular microscope. Preliminary drawings of cleared parts were traced from printed copies of images. Drawings were then edited by reference to the actual part.

Photomicrographs were taken with a Canon EOS 1000D digital SLR camera mounted on a Nikon SMZ800 binocular dissecting microscope equipped with a beam splitter. Measurements were made to the nearest 0.1 mm with the same microscope using an eyepiece grid and a reference scale. Photomicrographs used in the figures are focus-stacked composites prepared with Zerene Stacker 1.04.

Plates were composed using GIMP 2.8. Backgrounds in some photomicrographs have been edited to remove distracting highlights and artifacts. Maps were drawn with QGIS 2.4.

Latitude/longitude figures are given in decimal degrees to four decimal places, both in the text and in Suppl. material 1, together with an estimate of spatial uncertainty. In cases where label locality data are in the UTM system, both the two-letter, six-digit grid reference on the label (as formerly used in Tasmania) and its unambiguous, global UTM equivalent are provided in the text and in Suppl. material 1, together with the datum used.

### Abbreviations

AM
Australian Museum, Sydney, Australia 


MCZ
Museum of Comparative Zoology, Cambridge, USA 


MNHN
Muséum national d’Histoire naturelle, Paris, France 


QVM
 Queen Victoria Museum and Art Gallery, Launceston, Australia 


Tas
 Tasmania 


TMAG
Tasmanian Museum and Art Gallery, Hobart, Australia 


ZMB
Museum für Naturkunde, Berlin, Germany 


ZMUC
 Zoological collections of the Natural History Museum, Copenhagen, Denmark 


## Results

### Order Spirostreptida Brandt, 1833 Suborder Epinannolenidea Chamberlin, 1922 Family Iulomorphidae Verhoeff, 1924

#### Amastigogonus

Taxon classificationAnimaliaSpirostreptidaIulomorphidae

Brölemann, 1913

Amastigogonus Brölemann 1913: 152. Verhoeff 1924: 75, 84; 1932: 1732, 1737; 1944: 36, 41. Jeekel 1971: 107; 1981: 39; 2009: 35. Hoffman 1972: 204; 1980: 91. Mauriès 1987: 198. Mauriès et al. 2001: 585. Korsós and Johns 2009: 3. Edward and Harvey 2010: 5. Korsós and Read 2012: 44.
Euethogonus Chamberlin 1920: 166. Hoffman 1972: 204 (synonymised with Amastigogonus); 1980: 91.

##### Type species.

*Amastigogonus tasmanianus* Brölemann, 1913, by original designation.

##### Other assigned species.

*Amastigogonus danpicola* sp. n., *Amastigogonus elephas* sp. n., *Amastigogonus fossuliger* Verhoeff, 1944, *Amastigogonus hardyi* (Chamberlin, 1920), *Amastigogonus hellyeri* sp. n., *Amastigogonus michaelsae* sp. n., *Amastigogonus orientalis* sp. n., *Amastigogonus peninsulensis* sp. n., *Amastigogonus verreauxii* (Gervais, 1847).

##### Diagnosis.

Like *Victoriocambala* Verhoeff, 1944 in having the coxite process on the anterior gonopod close to the telopodite and nearly as long, forming a chamber resembling a bird’s beak in which the pseudoflagellum is protected. Differences between *Amastigogonus*/*Victoriocambala*, as noted by Jeekel (2009: 35), are leg 1 with free/fused tibia and tarsus, posterior gonopod without/with reduced telopodite.

##### Description.

Living animals usually with black or dark grey rings with annular pale band at rear of each metazonite, often with a greenish tinge (live *Amastigogonus fossuliger* more consistently green, see species description); head, collum and telson often faintly reddish brown; legs pale. With long storage in alcohol and staining by defensive secretion, animals dull grey with faintly reddish legs.

Observed midbody diameter of larger males 2.5-4.2 mm, 55-71 podous rings. Head smooth, slightly convex, vertigial sulcus reaching to level of dorsalmost ocellar row. Ocellar area of larger males lenticular with ca 25-50 ocelli in 4-6 somewhat irregular horizontal rows. Antennae short, barely reaching past posterior edge of collum when manipulated dorsally; relative antennomere lengths (2=3)>6>(4=5); antennomere 6 widest; 4 apical cones; socket ca 1 socket diameter from lateral margin of head capsule. Gnathochilarium with lateral edges of mentum slightly convex, mentum about as wide as combined lingual plates; mentum-promentum junction slightly concave anteriorly; a prominent pit with small seta anteriorly on each gnathochilarial stipes. Collum convex, laterally narrowing with rounded corner, margins straight. Prozonites only slightly narrower than metazonites; suture weakly defined; fine longitudinal striae on lower portion of metazonite, anterior end of each stria (Fig. 3C; s) bent obliquely upwards towards suture (see Remarks, below); prozonites and metazonites with surface otherwise smooth, free of setae. Ozopores (Fig. 3C; o) small, round, beginning ring 6 at a little over 1/2 ring height, ring 6 ozopore distinctly lower than ring 7 ozopore; each ozopore at ca 1/3 the distance from suture to posterior metazonite margin, and usually absent from apodous rings. Limbus lamellar, undivided. Preanal ring smooth, epiproct broadly rounded, extending slightly over anal valves; hypoproct with slightly convex margin.

**Figure 2.**
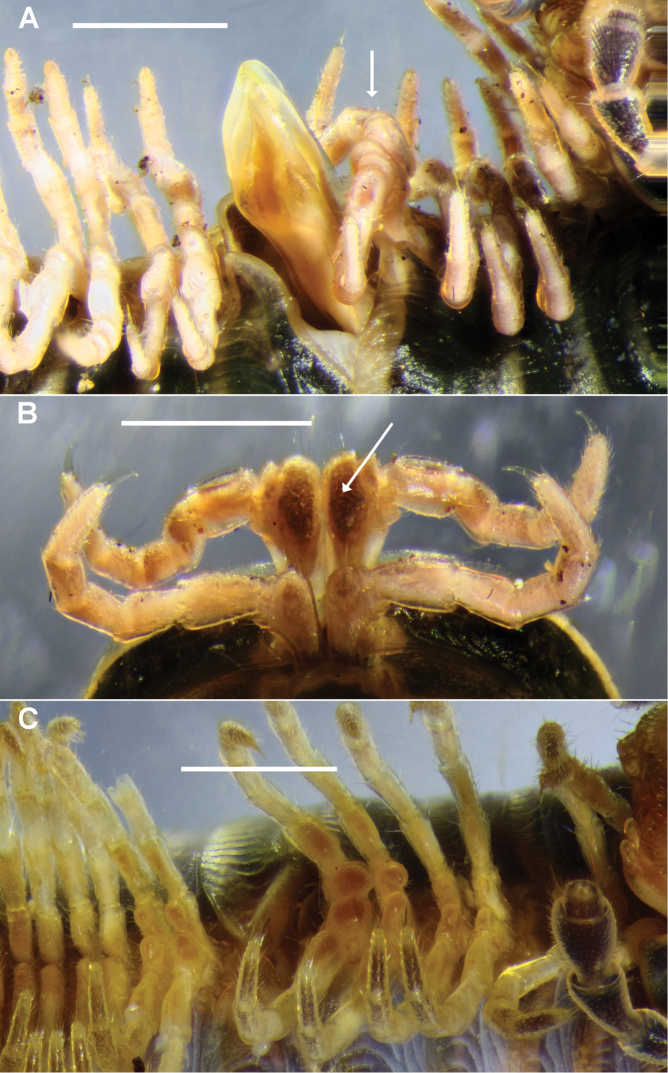
**A, B** *Amastigogonus fossuliger* Verhoeff, 1944 ex QVM 23:54468. **A** Left ventrolateral view of male with partly everted gonopods, showing legpair 7 (arrow) with elongated coxae **B** Anterior view of legpairs 6 and 7 on isolated ring 6 of same male, with leg 7 coxa marked with arrow **C** *Amastigogonus danpicola* sp. n., QVM 23:54403, left ventrolateral view of male. Scale bars = 1 mm.

Legpair 1 separate on coxosternite, each leg 1 with 5 podomeres without setae, anteroposteriorly somewhat flattened; relative podomere lengths typically femur>tibia>(prefemur=postfemur)>tarsus, relative widths typically prefemur> femur>tibia>postfemur>tarsus (see Remarks, below); no claw. Small brushes of setae on legpair 1 coxosternite anterior to and between legs, and laterally on coxosternite corners. Leg 2 leg-like with large claw and reduced prefemur; penis arising basally on posterior coxal surface, barrel-shaped with a few long setae in apical crown. Leg 7 (and sometimes other legs near gonopod aperture; see Remarks, below) with elongated coxa (Fig. 2). Midbody legs short, ca 2/3 ring diameter when extended; relative podomere lengths prefemur>(femur=tarsus)>(postfemur=tibia). Most prefemora distally with conical prefemoral pad (Fig. 5A, B; pa); pads first appear on ring 8 legs and diminish in size posteriorly; pads small or absent on last 2-4 legpairs (see Remarks, below).

**Figure 3.**
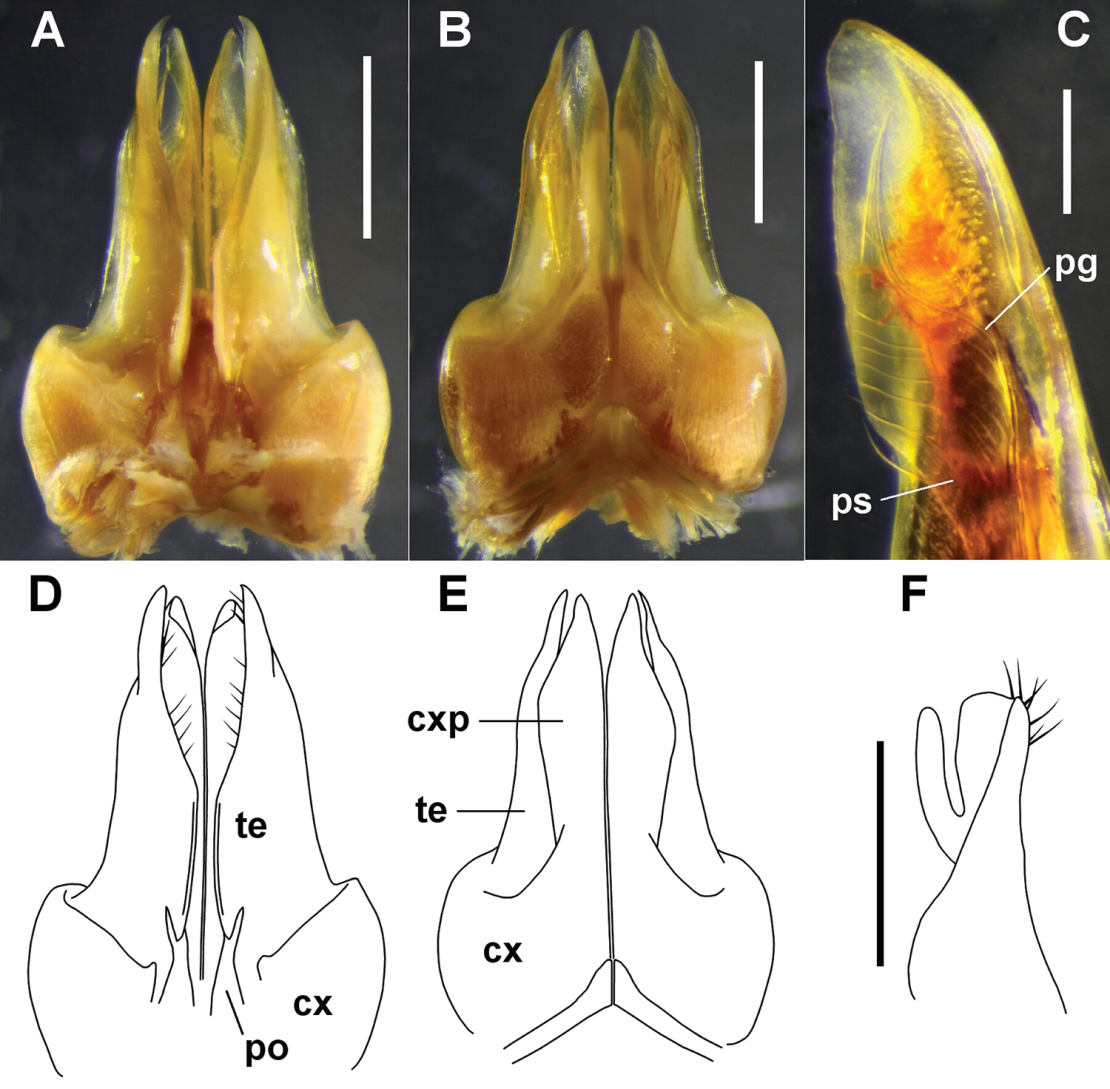
**A, B, D, E** *Amastigogonus hardyi* (Chamberlin, 1920) ex TMAG J5987. Posterior (**A, D**) and anterior (**B, E**) views of isolated gonopods, showing coxite (cx), coxite process (cxp) and telopodite (te) of anterior gonopod, and posterior gonopod (po) holding posteromedial flange of telopodite **C** *Amastigogonus fossuliger* Verhoeff, 1944, QVM 23:54356; anteromedial view of tip of anterior gonopod telopodite, showing transparent pseudoflagellum (ps) and prostatic groove (pg) **F** *Amastigogonus tasmanianus* (Chamberlin, 1920) ex QVM 23:54344, isolated right posterior gonopod, medial view. Scale bars: **A, B** = 1.0 mm, **C** = 0.25 mm, **F** = 0.5 mm.

Posterior margin of gonopod aperture raised and thickened on either side, adjoining tips of retracted anterior gonopods. Anterior gonopods (Figs 3A, 3B, 3D, 3E, 6, 8) parallel, closely appressed. Anterior gonopod coxite (Figs 3A, B, D, E; cx) massive, rounded laterally, extending thin, finger-shaped process (Figs 3A, 3B, 3D, 3E, 6, 8; cxp) from anteromedial surface, process slightly concave laterally, thickened medially to ca 2/3 process height (coxite process more complex in *Amastigogonus danpicola* sp. n., see species description). Telopodite (Figs 3A, 3B, 3D, 3E, 6, 8; te) arising from wide, shallow recess on coxite, paralleling coxite process and slightly longer; thin and slightly concave medially; somewhat thickened distally from near posterior margin to midline near apex, the thickening usually with row of more or less evenly spaced, prominent setae on posterior side and sometimes a separate row or group of setae on anterior side. Posterior surface of telopodite produced basally as rounded flange. Pseudoflagellum (Figs 3C, 6, 8; ps) thinly lamellar, usually arising at ca 1/2 telopodite height, usually supported by setae on distomedial surface of telopodite. Prostatic groove (Figs 3C, 6, 8; pg) running anterodistally from posterobasal corner of telopodite under rounded flange to pseudoflagellum, following anterior side of pseudoflagellum and terminating at pseudoflagellum tip. Posterior gonopod (Fig. 3F) ca 1/3 height of anterior gonopod, subcylindrical with apical recess posterolaterally, a crown of short setae around recess and an arm-like cylindrical process with rounded apex arising at ca 1/2 gonopod height on posterolateral surface and directed distally. In situ, posterior gonopod grips base of anterior gonopod telopodite between arm-like process and body of posterior gonopod, the posterior gonopod apex pressed against prostatic groove: “The rudimentary posterior gonopods (Fig. 3H) appear to be attached like forceps to the wall at the very base of the caudomedian ridges of the anterior gonopods (Fig. 3E)” (Mauriès, Golovatch and Hoffman 2001: 585).

Female slightly larger in diameter than male with same ring count; leg 1 normally leg-like, claw-bearing; no prefemoral pads on any legs.

##### Remarks.

**Figure 4.**
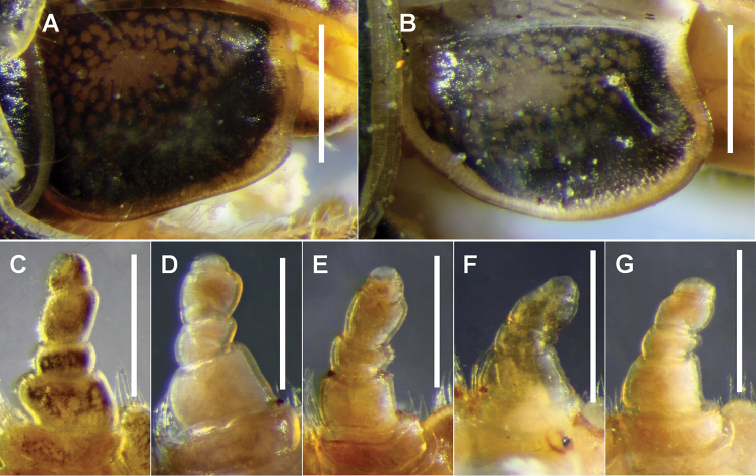
**A, B** Lateral view of right cardo of **A** *Amastigogonus danpicola* sp. n., holotype, QVM 23:54377 and **B** *Amastigogonus fossuliger* Verhoeff, 1944, ex QVM 23:54468 **C–G** Posterior view of left leg 1 of **C** *Amastigogonus danpicola* sp. n., ex QVM 23:54390 **D** *Amastigogonus hellyeri* sp. n., ex QVM 23:54470 **E** *Amastigogonus michaelsae* sp. n., QVM 23:54387 **F** *Amastigogonus tasmanianus* (Chamberlin, 1920), ex QVM 23:54344 and **G** *Amastigogonus verreauxii* (Gervais, 1847), TMAG J5915. All scale bars = 0.5 mm.

*Amastigogonus* species are closely similar in size, general appearance and habits, and males can only be positively identified by close inspection, and usually dissection, of the anterior gonopods. There is also some variation in non-gonopodal male structures, as noted here and in the species descriptions. The most reliable of these differences are in cardo shape and modifications of near-aperture legs:

*Cardo*. In *Amastigogonus danpicola* sp. n. the cardo extends further ventrally in its posterior half than in its anterior half, i.e. the cardo is deeper posteriorly (Fig. 4A). In other *Amastigogonus* species the ventral edge of the cardo is either evenly convex or is deeper anteriorly (Fig. 4B).

*Near-aperture legs*. In all *Amastigogonus* species, leg 7 has an elongated coxa (Fig. 2A, B; arrow). Less elongated coxae are also found on legs 10 and 11 in *Amastigogonus hellyeri* sp. n. and on legs 6, 10 and 11 in *Amastigogonus danpicola* sp. n. (Fig. 2C).

Other differences between species do not seem to be large enough or consistent enough to be useful for taxonomic purposes:

*Legpair 1*. The relative lengths and widths of the leg 1 podomeres vary a little between species (Fig. 4C–G), between individuals, and sometimes between right and left legs.

**Figure 5.**
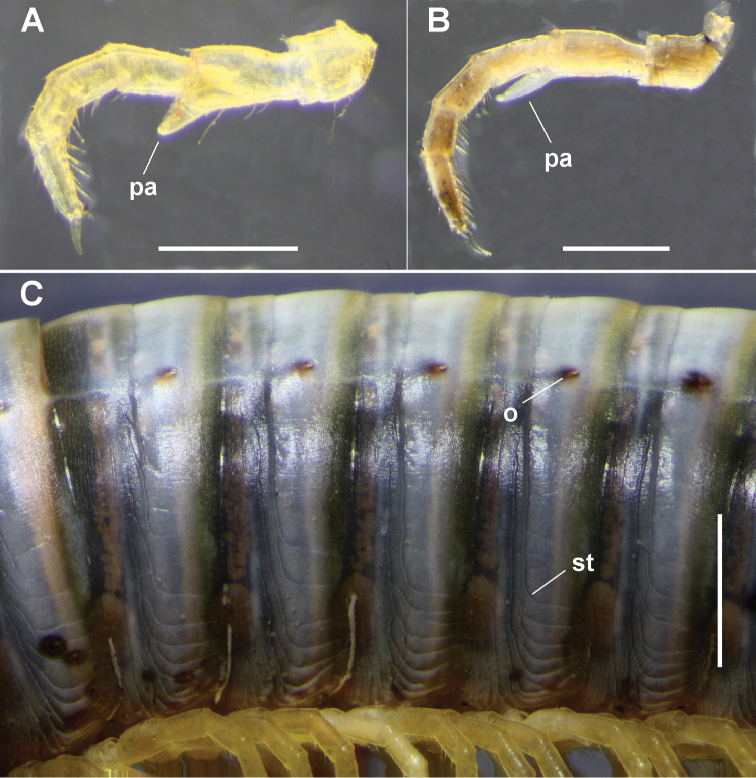
**A, B** Male midbody leg of **A** *Amastigogonus tasmanianus* (Chamberlin, 1920), ex QVM 23:54344 and **B** *Amastigogonus fossuliger* Verhoeff, 1944, ex QVM 23:54468, showing prefemoral pad (pa) **C** *Amastigogonus hardyi* (Chamberlin, 1920), male ex TMAG J5897, left lateral view of rings at ca 2/3 body length, showing position of topmost horizontal stria (st) relative to ozopore (o). Scale bars: **A, B** = 0.5 mm, **C** = 1.0 mm.

*Prefemoral pads*. There are differences between species in pad length, as shown in Fig. 5A and 5B (pa), but these differences are masked by the anteroposterior size gradient on single individuals as well as by variability from individual to individual.

*Metazonal striae*. The height reached by the horizontal striae on the metazonites diminishes slightly from anterior to posterior. At ca 2/3 body length, the topmost horizontal stria lies at ca 1/2 or ca 3/4 of the height to the ozopore (Fig. 5C; st), depending on species, but with considerable variation between rings and between individuals.

#### Amastigogonus danpicola sp. n.

Taxon classificationAnimaliaSpirostreptidaIulomorphidae

http://zoobank.org/A2FB1293-9FCD-4F34-B8E3-DA5F9160C4EF

**Figure 6.**
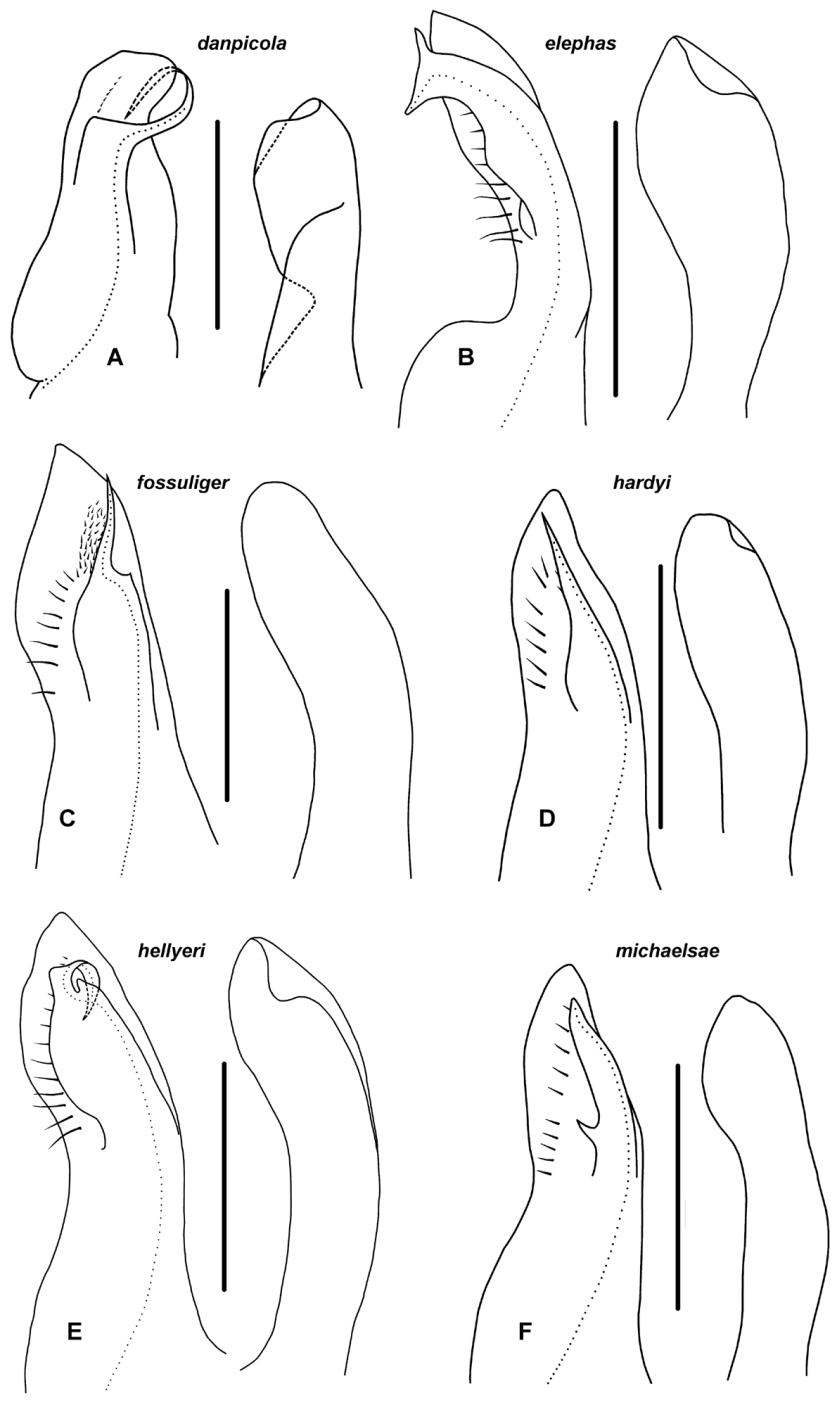
Right anterior gonopod, medial view of distal portion of telopodite (left) and lateral view of distal portion of coxite process (right); fo = fossa, cxp = coxite process, fl = posterobasal flange on telopodite, pg = prostatic groove, ps = pseudoflagellum, t = folded-over tab on coxite process, te = telopodite. Scale bars = 1 mm; dotted line indicates course of prostatic groove. **A** *Amastigogonus danpicola* sp. n., ex QVM 23:54378 **B** *Amastigogonus elephas* sp. n., paratype, QVM 23:54369 **C** *Amastigogonus fossuliger* Verhoeff, 1944, QVM 23:54406 **D** *Amastigogonus hardyi* (Chamberlin, 1920), TMAG J5987 **E** *Amastigogonus hellyeri* sp. n., paratype ex QVM 23:54515 **F** *Amastigogonus michaelsae* sp. n., QVM 23:54387.

Figs 2C , 4A , 4C , 6A

##### Holotype.

Male, Apsley River, Tas, -41.7992 148.1508 ±250 m [label “EP 955 717” (= 55G 595500 5371700, AGD66)], 300 m a.s.l., 5 July 1988, R. Mesibov, QVM 23:54377 (specimen in 3 pieces).

##### Paratypes.

1 male, locality and collector as for holotype but -41.7972 148.1544 ±250 m [label “EP 958 719” (= 55G 595800 5371900, AGD66)], 320 m a.s.l., 6 July 1988, R. Mesibov, QVM 23:54371; 1 male, same locality and collector but -41.7964 48.1592 ±250 m [label “EP 962 720” (= 55G 596200 5372000, AGD66)], 350 m a.s.l., 18 July 1988, R. Mesibov, QVM 23:54373.

##### Other material.

22 males, 4 probable females from 16 unique localities; details in Suppl. material 1.

##### Diagnosis.

Coxite process of the anterior gonopod divided by narrow fossae apically and anterobasally; legs 6, 7, 10 and 11 with elongated coxae.

##### Description.

Mature males observed with (48+4) rings, 2.6 mm midbody diameter to (71+1) rings, 3.2 mm. Cardo deeper posteriorly than anteriorly (Fig. 4A; ce). Legs 6, 7, 10 and 11 with elongated coxae, swollen distomedially (Fig. 2C); leg 7 coxa longest and most swollen. Prefemoral pad ca 3/4 femur length. Striae on posterior metazonites reaching ca 1/2 ozopore height.

Coxite process on anterior gonopod (Fig. 6A; cxp) with basal portion divided anteriorly by fossa (Fig. 6A; fo) into lateral and medial longitudinal flanges, the medial flange with deep, V-shaped notch at ca 1/2 coxite height on anterior margin; process divided apically by shallow fossa (Fig. 6A; fo) separating distolateral and distomedial margins; process not reaching level of telopodite apex. Telopodite (Fig. 6A; te) with row of minute setae on posterior side of medial thickening, behind pseudoflagellum (Fig. 6A; ps). Pseudoflagellum ca 1/2 telopodite width at base, truncate at ca 1/2 pseudoflagellum height, extending anterodistally from anterior corner of truncate basal portion as thin, flexible ribbon, the latter usually curving laterally over telopodite apex in preserved specimens.

##### Distribution.

**Figure 7.**
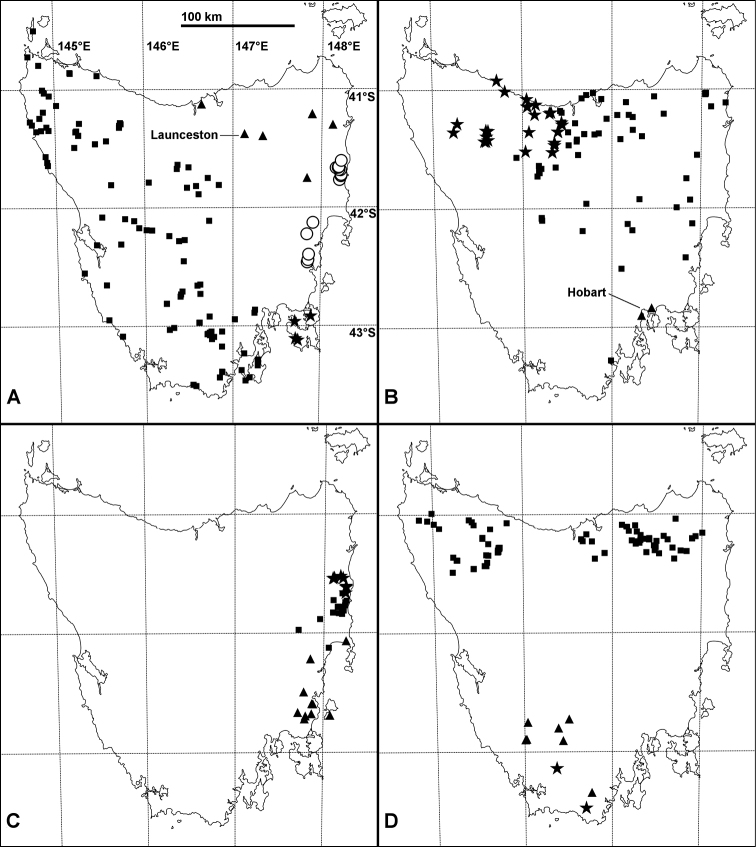
Known localities as of 14 July 2016 for Tasmanian Iulomorphidae; Mercator projections with approximate distance scale. **A** *Amastigogonus orientalis* sp. n. (open circles), *Amastigogonus peninsulensis* sp. n. (stars), *Amastigogonus tasmanianus* Brölemann, 1913 (triangles), *Amastigogonus verreauxii* (Gervais, 1847) (squares) **B** *Amastigogonus fossuliger* Verhoeff, 1944 (squares), *Amastigogonus hardyi* (Chamberlin, 1920) (triangles), *Amastigogonus hellyeri* sp. n. (stars) **C** *Amastigogonus danpicola* sp. n. (squares), *Amastigogonus elephas* sp. n. (stars), *Amastigogonus michaelsae* sp. n. (triangles) **D** *Atelomastix bonhami* sp. n. (triangles), *Atelomastix smithi* sp. n. (stars), *Equestrigonus tasmaniensis* gen. n., sp. n. (squares).

Eucalypt forest over ca 1000 km^2^ on the East Coast of Tasmania, mainly in the Apsley, Douglas, St Pauls and Swan River catchments (Fig. 7C), from near sea level to at least 600 m. Possibly parapatric with *Amastigogonus elephas* sp. n. in the upper St Pauls River catchment and with *Amastigogonus michaelsae* sp. n. near Swansea. Sympatric with *Amastigogonus fossuliger* and with *Amastigogonus orientalis* sp. n.

##### Name.

Abbreviation in lower case “danp” for Douglas-Apsley National Park plus Latin *cola*, inhabitant; noun in apposition. This species is abundant in the Park, which also contains the type locality.

##### Remarks.

*Amastigogonus danpicola* sp. n. is the most apomorphic species within the group included here in *Amastigogonus*. I place it in this genus because the structure and position of the pseudoflagellum and its supporting setae conform to the general pattern seen in the other *Amastigogonus* species.

#### Amastigogonus elephas sp. n.

Taxon classificationAnimaliaSpirostreptidaIulomorphidae

http://zoobank.org/0F43E23A-AA7E-4356-8AB7-891A95DC64FA

Fig. 6B

##### Holotype.

Male, Mt Elephant, Tas, -41.6338 148.2421 ±25 m, 420 m a.s.l., 13 May 2016, R. Mesibov, QVM 23:54519.

##### Paratypes.

3 males , 3 females, details as for holotype, QVM 23:54520; 1 male, same locality but -41.6244 148.2425 ±250 m [label “FP 034 910” (= 55G 603400 5391000, AGD66)], 19 January 2002, R. Mesibov and T. Moule, QVM 23:54369.

##### Other material.

6 males from 5 unique localities; details in Suppl. material 1.

##### Diagnosis.

Like *Amastigogonus verreauxii* in having a telopodite with a subquadrate extension of the posterobasal margin and a posteriorly curving pseudoflagellum; distinguished from *Amastigogonus verreauxii* in the pseudoflagellum having a small, tooth-like, distally directed extension basal to the posterobasally directed tip.

##### Description.

Mature males observed with (47+3) rings, 3.2 mm midbody diameter to (69+0) rings, 3.6 mm. Cardo not deeper posteriorly. Leg 7 (only) with elongated coxa. Prefemoral pad ca 1/2 femur length. Striae on posterior metazonites reaching ca 3/4 of ozopore height.

Coxite process on anterior gonopod (Fig. 6B) with small portion of posterodistal margin extended as rounded tab (Fig. 6B; t), sharply folded over laterally; process nearly reaching level of telopodite apex. Telopodite with posterobasal surface produced posteriorly as subquadrate flange (Fig. 6B; fl) and with single row of prominent setae on posterior side of medial thickening. Pseudoflagellum with small rounded swelling adjoining base posteromedially; arising at 1/3–1/2 telopodite height, ca 2/3 width of telopodite at base, slightly curving posteriorly, slightly expanded posteriorly at 1/3–1/2 pseudoflagellum height, apically with small, rounded, distally directed, tooth-like extension, the prostatic groove opening on posterobasally directed pseudoflagellum tip.

##### Distribution.

Eucalypt forest at the eastern end of the Fingal Valley on the Tasmanian East Coast (Fig. 7C), to at least 700 m elevation. The small range of this species is home to other locally endemic invertebrates, including the millipede *Tasmaniosoma nicolaus* Mesibov, 2015 and the onychophoran *Tasmanipatus anophthalmus* Ruhberg, Mesibov, Briscoe & Tait, 1991. *Amastigogonus elephas* sp. n. may be parapatric with *Amastigogonus danpicola* sp. n. in the upper St Pauls River catchment.

##### Name.

Latin *elephas*, elephant; noun in apposition. For the type locality, Mt Elephant.

##### Remarks.

**Figure 8.**
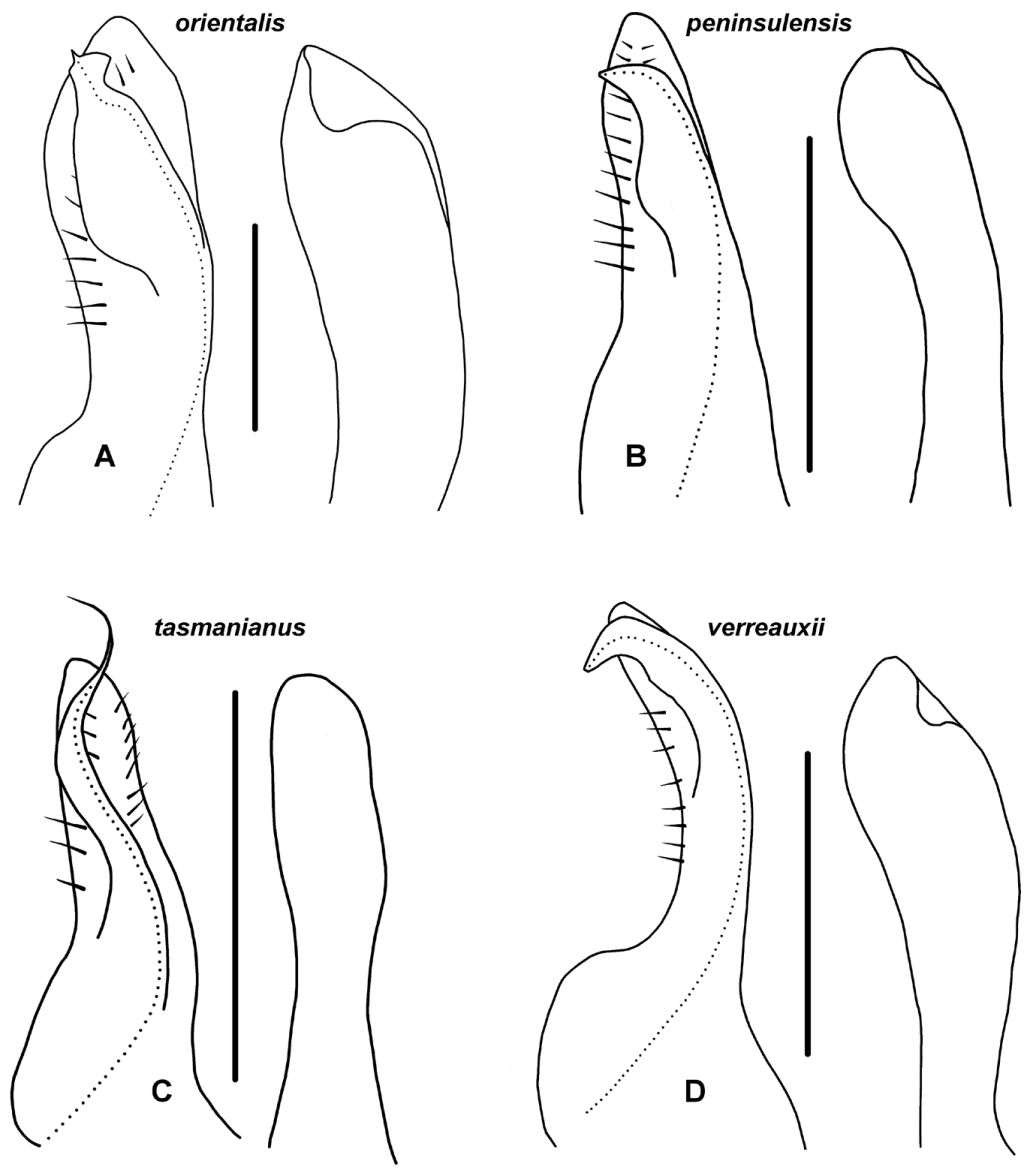
Right anterior gonopod, medial view of distal portion of telopodite (left) and lateral view of distal portion of coxite process (right); cxp = coxite process, fl = posterobasal flange on telopodite, pg = prostatic groove, ps = pseudoflagellum, t = folded-over tab on coxite process, te = telopodite. Scale bars = 1 mm; dotted line indicates course of prostatic groove. **A** *Amastigogonus orientalis* sp. n., paratype ex QVM 23:54401 **B** *Amastigogonus peninsulensis* sp. n., QVM 23:54474 **C** *Amastigogonus tasmanianus* Brölemann, 1913, ex QVM 23:54469 **D** *Amastigogonus verreauxii* (Gervais, 1847), TMAG J5915.

The anterior gonopod of *Amastigogonus elephas* sp. n. (Fig. 6B) is similar to that of *Amastigogonus verreauxii* (Fig. 8D). The wide disjunction in the species ranges (more than 100 km) and the remarkable consistency of form in the anterior gonopod of *Amastigogonus verreauxii* (across a linear range extent of ca 400 km) suggests that the two lineages have long been separated.

#### Amastigogonus fossuliger

Taxon classificationAnimaliaSpirostreptidaIulomorphidae

Verhoeff, 1944

Figs 1 , 2A, B , 3C , 4B , 5B , 6C

Amastigogonus fossuliger Verhoeff 1944: 43, figs 6–8. Jeekel 1981: 43. Korsós and Read 2012: 45.

##### Syntypes.

At least 1 male and 1 female, Lake Leake, Tasmania, date and collector unknown (see Remarks, below), specimens not located.

##### Other material.

130 males and 12 females from 66 unique localities; details in Suppl. material 1.

##### Diagnosis.

Coxite process of anterior gonopod with posterodistal margin not extended; pseudoflagellum with dense field of short setae on telopodite behind pseudoflagellum tip; pseudoflagellum with distinct shoulder, the prostatic groove making an S-bend before entering the abruptly tapered tip of the pseudoflagellum.

##### Description.

Live males and females with more or less greenish-grey rings (Fig. 1A, C). Mature males observed with (39+4) rings, 2.1 mm midbody diameter to (63+1) rings, 3.6 mm. Cardo not deeper posteriorly (Fig. 4B). Leg 7 (only) with elongated coxa (Fig. 2A, C). Prefemoral pad ca 3/4 or more femur length (Fig. 5B). Striae on posterior metazonites reaching ca 2/3-3/4 of ozopore height.

Coxite process on anterior gonopod (Fig. 6C) with posterodistal margin not extended and folded over laterally. Telopodite with single row of prominent setae on posterior side of medial thickening to near telopodite apex, the thickening then widening and bearing dense brush of numerous minute setae. Pseudoflagellum ca 1/2 width of telopodite at base, tapering abruptly at ca 1/2 pseudoflagellum height to narrow, sharply pointed tip with rounded shoulder on anterior side of tapered section (Fig. 3C; ps), sometimes with small, tooth-like, anterodistal extension on shoulder; prostatic groove (Fig. 3C; pg) making S-bend from anterior side of pseudoflagellum into tapered tip.

##### Distribution.

Widespread in the eastern half of Tasmania (Fig. 7B) in dry and wet eucalypt forest from near sea level to at least 1050 m, extending across the Central Plateau to the Cradle Mountain area. Parapatric with *Amastigogonus hellyeri* sp. n. along the Mersey Break, a well-documented faunal divide for millipedes in north central Tasmania (Mesibov 1999). Co-occurs with *Amastigogonus tasmanianus* in northeast Tasmania. Overlaps with *Amastigogonus verreauxii* on the Central Plateau and possibly in southern Tasmania, and to a small extent with *Amastigogonus danpicola* sp. n. southeast of the Fingal Valley. The far southern record on the distribution map (Fig. 7B) is for two *Amastigogonus fossuliger* males found on the verandah of a house at Francistown, and may represent an accidental translocation rather than a natural occurrence.

##### Remarks.

At least one male and one female of this species from the Lake Leake area were probably sent to Verhoeff by George Edward Nicholls, a Western Australian biologist who collected in Tasmania in 1928, 1929 and 1939 (Nicholls 1943). Verhoeff (1936: 14) had previously thanked Nicholls for providing specimens of an unrelated millipede species from Lake Leake.

I have trouble understanding the differences in the two anterior gonopods illustrated by Verhoeff (1944), both presumably from Lake Leake specimens and possibly from the same male. Verhoeff’s fig. 6 shows a right gonopod tip in posterior view, fig. 7 a left gonopod tip in medial view. The thread-like pseudoflagellum in fig. 6 has a tooth-like extension on the shoulder and an intact posterobasal margin, while the shorter, tapered pseudoflagellum in fig. 7 has no tooth-like extension and a notched posterobasal margin. The remarkable thinness and fragility of the *Amastigogonus fossuliger* pseudoflagellum may be the explanation for Verhoeff’s difficulties in seeing and drawing these features. My Fig. 6C is based on a male from near the type locality, while the image in Fig. 3C is of a male from Ansons Bay, ca 100 km to the north. The only significant difference is the absence of a tooth-like extension in the latter.

*Amastigogonus fossuliger* is more consistently and more obviously green than other *Amastigogonus* species, but the green colour varies in intensity from individual to individual.

#### Amastigogonus hardyi

Taxon classificationAnimaliaSpirostreptidaIulomorphidae

(Chamberlin, 1920)

Figs 3A, B, D, E , 5C , 6D

Euethogonus hardyi Chamberlin 1920: 166. Jeekel 1971: 109.
Amastigogonus hardyi Hoffman 1972: 204 (new combination), figs 6–8. Jeekel 1981: 43. Korsós and Read 2012: 45.
Amastigogonus nichollsii Verhoeff 1944: 43, figs 1–5. Hoffman 1972: 204 (synonymised with Amastigogonus hardyi).

##### Holotype of *Euethogonus hardyi*.

Male, Tasmania, date unknown, G.H. Hardy, MCZ 4817. Illustrated by Hoffman (1972); not examined.

##### Paratypes of *Euethogonus hardyi*.

At least 1 female, details as for holotype, MCZ 4818. Not examined.

##### Lectotype of *Amastigogonus nichollsii*

**(here designated).** Male (slide mount of gonopods), Mt Nelson, Tasmania, date and collector unknown, ZMB 12642a (= Verhoeff collection slide 3777). Illustrated by Verhoeff (1944); material examined as image.

##### Paralectotypes of *Amastigogonus nichollsii*.

1 female, same collection details, ZMB 12642; not examined. Verhoeff (1944: 44) describes a second male specimen, but this has not been located; this male is another paralectotype.

##### Other material.

3 males and 3 females from 2 unique localities; details in Suppl. material 1.

##### Diagnosis.

Like *Amastigogonus fossuliger* in having the pseudoflagellum with a narrow, tapering, pointed tip; distinguished by the prostatic groove following a straight course on the pseudoflagellum rather than having an S-bend, and by the lack of a dense field of short setae on the telopodite behind the pseudoflagellum tip.

##### Description.

Three mature males examined: (52+3) rings, 2.8 mm midbody diameter, (61+1) rings, 3.1 mm and (65+1) rings, 3.2 mm. Cardo not deeper posteriorly. Leg 7 (only) with elongated coxa. Prefemoral pad ca 1/2 femur length. Striae on posterior metazonites reaching ca 1/2 of ozopore height (Fig. 5C).

Coxite process on anterior gonopod (Fig. 6D) with small portion of posterodistal margin extended as rounded tab and folded over laterally. Telopodite with single row of prominent setae on posterior side of medial thickening and short row on anterior side near apex. Pseudoflagellum ca 1/2 telopodite width at base, slightly extended posteriorly at base, then gradually tapering to sharp, posterodistally directed point.

##### Distribution.

Currently known only from eucalypt forest at two sites in the Hobart metropolitan area (Fig. 7B) in Tasmania: Mt Nelson (including 1973 collections at this type locality for *Amastigogonus nichollsii*) and Mt Rumney.

##### Remarks.

The types of *Euethogonus hardyi* were collected by the entomologist George H. H. Hardy, probably during Hardy’s tenure as Assistant Curator of the Tasmanian Museum in Hobart, 1913-1917 (Marks 1991: 216). Hoffman (1972) examined and illustrated the *Euethogonus hardyi* holotype and assigned the species to *Amastigogonus*. The locality given for the types is simply “Tasmania” (Hoffman 1972: 204). Chamberlin (1920: 166-167) did not specify the number or gender of the *Euethogonus hardyi* paratypes.

Two males and one female of *Amastigogonus nichollsii* were presumably sent to Verhoeff by G.E. Nicholls, collector of the *Amastigogonus fossuliger* types. Hoffman (1972) synonymised *Amastigogonus nichollsii* with *Amastigogonus hardyi* after comparing Verhoeff’s drawings of slide-mounted *nichollsii* gonopods with the unmounted gonopods of the *hardyi* holotype. After examining males from the *nichollsii* type locality, Mt Nelson (TMAG J5897 and J5926), I agree with Hoffman that *nichollsii* is a junior synonym of *hardyi*.

#### Amastigogonus hellyeri sp. n.

Taxon classificationAnimaliaSpirostreptidaIulomorphidae

http://zoobank.org/FF20C7F6-F918-4BD8-B912-F25F8BDF1EF2

Figs 4D , 6E

##### Holotype.

Male, Keddies Creek area, Tas, -41.1704 146.0545 ±25 m, 60 m a.s.l., 7 May 2016, R. Mesibov, QVM 23:54471.

##### Paratypes.

1 male, details as for holotype, QVM 23:54472; 2 males, same locality and collector but -41.1672 146.0569 ±250 m, 50-120 m a.s.l., 11 May 2016, tree trunks along Dial Road at night, QVM 23:54515.

##### Other material.

142 males and 10 females from 26 unique localities; details in Suppl. material 1.

##### Diagnosis.

Like *Amastigogonus orientalis* sp. n. in having a broad pseudoflagellum abruptly reduced apically; distinguished by having a smoothly curving rather than a subquadrate extension of the posterobasal telopodite margin, with a notch anteriorly at the base of the reduced pseudoflagellum tip and the tip relatively long and curving laterally or medially.

##### Description.

Mature males observed with (40+4) rings, 2.5 mm midbody diameter to (63+1) rings, 3.9 mm. Cardo not deeper posteriorly. Leg 7 with elongated coxa, legs 10 and 11 with less elongated coxae. Prefemoral pad ca 1/2 femur length. Striae on posterior metazonites reaching ca 3/4 of ozopore height.

Coxite process on anterior gonopod (Fig. 6E) with posterodistal margin extended as rounded tab and sharply folded over laterally. Telopodite with single row of prominent setae on posterior side of medial thickening. Pseudoflagellum ca 2/3 telopodite width at base, posterior margin sometimes sinuous in transverse plane; abruptly narrowing apically with a shallow notch dividing the apex into small, rounded, anterior tooth and short, thinly laminar, acutely pointed extension, the latter usually folded over laterally and carrying the prostatic groove to its pointed tip.

##### Distribution.

Eucalypt forest and cool temperate rainforest in northwest Tasmania (Fig. 7B), from near sea level to at least 760 m. Meets *Amastigogonus fossuliger* parapatrically along the Mersey Break (Mesibov 1999) and overlaps to a small extent in far northwest Tasmania with *Amastigogonus verreauxii*.

##### Name.

For Henry Hellyer (1790-1832), explorer of northwest Tasmania; noun in the genitive case.

##### Remarks.

The tip of the pseudoflagellum in preserved males is often bent laterally into the space between the pseudoflagellum and the rest of the telopodite, as shown in Fig. 6E. In other specimens it may be bent medially towards the coxite tip, or extend past the tip of the telopodite. This species otherwise varies little across its range.

#### Amastigogonus michaelsae sp. n.

Taxon classificationAnimaliaSpirostreptidaIulomorphidae

http://zoobank.org/EACF58B3-B650-4595-ADE9-69F25812F1EA

Figs 4E , 6F

##### Holotype.

Male, Douglas Creek, Tas, -42.5139 147.7767 ±100 m [label “EN 637 927” (= 55G 563700 5292700, AGD66)], 210 m a.s.l., 24 April 1991, R. Mesibov, QVM 23:54374; dissected, with head and anterior rings in genitalia vial.

##### Paratypes.

2 males, Montgomery Road, Tas, -42.6863 147.7111 ±50 m, 330 m a.s.l., 21 June 2016, R. Mesibov, QVM 23:54548.

##### Other material.

11 males and 1 probable female from 8 unique localities; details in Suppl. material 1.

##### Diagnosis.

Like *Amastigogonus peninsulensis* sp. n. in having a relatively broad, gently tapering pseudoflagellum; distinguished by the pseudoflagellum tip directed distally rather than posteriorly and with a prominent, posterodistally directed tooth basally on the posterior margin.

##### Description.

Mature males observed with (55+1) rings, 3.3 mm midbody diameter to (66+1) rings, 3.4 mm. Cardo not deeper posteriorly. Leg 7 (only) with elongated coxa. Prefemoral pad ca 3/4 femur length. Striae on posterior metazonites reaching 3/4 ozopore height.

Coxite process on anterior gonopod (Fig. 6F) with posterodistal margin not extended and folded over. Telopodite without pronounced medial thickening, but with usual row of setae from near posterior margin to midline at telopodite apex, continued basally as group of sparse setae (not shown in Fig. 6F) behind tip of pseudoflagellum. Pseudoflagellum ca 1/3 telopodite width at base, curving slightly posteriorly and tapering gradually to rounded, posteriorly directed apex, and with short, sharp, posterodistally directed tooth at ca 1/4 pseudoflagellum height on posterior margin.

##### Distribution.

Eucalypt forest in southeast Tasmania from Coles Bay south to the Nugent area, including Maria Island (Fig. 7C), from sea level to at least 590 m. Possibly parapatric with *Amastigogonus danpicola* sp. n. near Swansea, parapatric or overlapping with *Amastigogonus orientalis* sp. n. west of Triabunna.

##### Name.

For the ecologist Karyl Michaels, who trapped specimens in the previously little-sampled dry forests of southeast Tasmania; noun in the genitive case.

##### Remarks.

Most of the non-type males are partial or fragmented specimens.

#### Amastigogonus orientalis sp. n.

Taxon classificationAnimaliaSpirostreptidaIulomorphidae

http://zoobank.org/8DAE3EF0-0CBE-4EEA-AF03-BFF855C47EA5

Fig. 8A

##### Holotype.

Male, Maclaines Creek, Tas, -42.4628 147.8564 ±100 m [label “EN 703 983” (= 55G 570300 5298300, AGD66)], 260 m a.s.l., 26 April 1991, R. Mesibov, QVM 23:54523 (in 2 pieces).

##### Paratypes.

2 males, details as for holotype, QVM 23:54401.

##### Other material.

14 males and 3 probable females from 14 unique localities; details in Suppl. material 1.

##### Diagnosis.

Like *Amastigogonus hellyeri* sp. n. in having a broad pseudoflagellum abruptly narrowed apically; distinguished by having a subquadrate extension of the posterobasal telopodite margin, with the prostatic groove opening on a very short tooth-like extension of the reduced pseudoflagellum tip, rather than on a relatively long, flexible extension.

##### Description.

Mature males observed with (43+3) rings, 2.6 mm midbody diameter to (67+0) rings, 4.2 mm. Cardo not deeper posteriorly. Leg 7 (only) with elongated coxa. Prefemoral pad ca 1/2 femur length. Striae on posterior metazonites reaching 3/4 ozopore height.

Coxite process on anterior gonopod (Fig. 8A) with posterodistal margin substantially extended as rounded tab and folded over laterally to ca 1/2 process width. Telopodite with posterobasal surface produced posteriorly as large rounded flange (Fig. 8A; fl) and with single row of prominent setae on posterior side of medial thickening. Pseudoflagellum ca 1/2 width of telopodite at base, expanded slightly posteriorly, tapering gradually before expanding distally in wedge shape, often curving laterally, with very short pointed extension at middle of distal margin; prostatic groove making S-bend from anterior side of pseudoflagellum into tip, terminating in pointed extension. Posterior margin of pseudoflagellum sometimes sinuous in transverse plane.

##### Distribution.

Eucalypt forest over ca 100 km linear extent in the Eastern Tiers of Tasmania (Fig. 7A) from ca 150 to at least 600 m elevation; the disjunction in the distribution map is likely to be a sampling artefact. Possibly parapatric with *Amastigogonus elephas* sp. n. near Gray; overlapping to a small extent with *Amastigogonus danpicola* sp. n. in the Douglas-Apsley National Park and with *Amastigogonus fossuliger* northwest of Triabunna; parapatric or overlapping with *Amastigogonus michaelsae* sp. n. in the southern Eastern Tiers.

##### Name.

Latin *orientalis*, eastern; adjective. This species is restricted to the East Coast region of Tasmania.

#### Amastigogonus peninsulensis sp. n.

Taxon classificationAnimaliaSpirostreptidaIulomorphidae

http://zoobank.org/2EB82B9E-9B71-4B83-864D-AE2AA906DBAF

Fig. 8B

##### Holotype.

Male, Coal Mine Hill, Tas, -42.9852 147.7113 ±25 m, 40 m a.s.l., 20 June 2016, R. Mesibov, QVM 23:54544.

##### Paratypes.

13 males and 5 females, details as for holotype, QVM 23:54545.

##### Other material.

3 males and 1 female from 3 unique localities; details in Suppl. material 1.

##### Diagnosis.

Like *Amastigogonus michaelsae* sp. n. in having a relatively broad, gently tapering pseudoflagellum, but with the tip directed distally and without a prominent tooth on the posterior margin.

##### Description.

Mature males observed with (40+3) rings, 1.8 mm midbody diameter to (60+1) rings, 2.5 mm. Cardo not deeper posteriorly. Leg 7 (only) with elongated coxa. Prefemoral pad ca 3/4 femur length. Striae on posterior metazonites reaching 1/2 ozopore height.

Coxite process on anterior gonopod (Fig. 8B) with very small portion of posterodistal margin slightly extended as rounded tab, bent laterally rather than folded over. Telopodite with single row of prominent setae on posterior side of medial thickening and shorter row of similarly prominent setae on anterior side. Pseudoflagellum ca 1/2 width of telopodite at base, posterior margin a little expanded at 1/3-1/2 pseudoflagellum height, apex acuminate and directed posteriorly.

##### Distribution.

Eucalypt forest on Forestier and Tasman Peninsulas (Fig. 7A) in Tasmania, from near sea level to at least 260 m.

##### Name.

For the Tasman Peninsula, type locality of this species; adjective.

#### Amastigogonus tasmanianus

Taxon classificationAnimaliaSpirostreptidaIulomorphidae

Brölemann, 1913

Figs 3F , 4F , 5A , 8C

Amastigogonus tasmanianus Brölemann 1913: 153, figs 32–37. Attems 1914: 293. Chamberlin 1920: 167. Verhoeff 1944: 43. Jeekel 1971: 107; 1981: 40. Korsós and Read 2012: 45.

##### Lectotype

**(here designated).** Male, Tasmania, date and collector unknown, AM KS.125304 (ex KS.37403). Intact specimen in 12 mm glass vial in 80% alcohol with original label “Amastigogonus / tasmanianus m. / (H.W.B.)”.

##### Paralectotypes.

Collection details as for lectotype, AM KS.37403. 1 female and parts of 2 dissected males: last 23 podous rings + apodous rings + telson, midbody 31 rings, head capsule + collum, and ring 7 (gonopods missing) and following 2 podous rings. Also 8 small filter-paper envelopes containing cleared body parts illustrated by Brölemann (1913). In 25 mm glass vial with original label “Amastigogonus / tasmanianus m. / (H.W.B.)”.

##### Other material.

92 males, 1 probable female and 1 possible juvenile from 8 unique localities; details in Suppl. material 1.

##### Diagnosis.

Distinguished from all other *Amastigogonus* species by having a long, gradually tapering, narrowly ribbon-like pseudoflagellum.

##### Description.

Mature males observed with (46+2) rings, midbody diameter 2.5 mm to (67+1) rings, 3.4 mm. Cardo not deeper posteriorly. Leg 7 (only) with elongated coxa. Prefemoral pad 1/3-1/2 femur length (Fig. 5A). Striae on posterior metazonites reaching ca 1/2 of ozopore height.

Coxite process on anterior gonopod (Fig. 8C) with posterodistal margin not extended and folded over. Telopodite with single row of prominent setae on posterior and anterior sides of medial thickening. Pseudoflagellum arising at 1/3-1/2 telopodite height, ca 1/2 width of telopodite at base, ribbon-like, curving sinuously first posterodistally, then anterodistally, tapering abruptly to sharp point (ribbon-like apex of pseudoflagellum shown edge-on in Fig. 8C).

##### Distribution.

Eucalypt forest and cool temperate rainforest at scattered locations in northeast Tasmania (Fig. 7A), from ca 100 m elevation to at least 1000 m. Co-occurs with *Amastigogonus fossuliger*.

##### Remarks.

The types were in excellent condition when examined in 2016. Because it is not possible to decide which of the two dissected male syntypes (or both) was illustrated by Brölemann, I am unable to follow Recommendation 74B (Preference for illustrated specimen) of the International Code of Zoological Nomenclature in choosing a lectotype, and instead have selected the intact male syntype. The lectotype is the third of the three males listed by Brölemann (1913: 154): “length 44 m/m; diameter 2.80 m/m; 56 segments; three segments apodous; 99 pair of legs”.

I suspect that the type locality is the Launceston area, and the QVM male illustrated in Figs 3F, 4F and 5A is from Mowbray in Launceston.

#### Amastigogonus verreauxii

Taxon classificationAnimaliaSpirostreptidaIulomorphidae

(Gervais, 1847)

Figs 4G , 8D

Iulus verreauxii Gervais 1847: 175.
Julus Verreauxii Preudhomme de Borre 1884: 62.
“Julus” verreauxii Jeekel 1981: 43.
Amastigogonus verreauxii Mauriès et al. 2001: 585 (new combination), fig. 3. Korsós and Read 2012: 45.

##### Holotype.

Male, “De la Nouvelle-Hollande, sur le penchant du mont Wellington, par M. Jules Verreaux” (Gervais 1847: 175), MNHN GA031. Described and illustrated by Mauriès et al. (2001), specimen not re-examined.

##### Other material.

360 males, 100 probable females and 34 possible juveniles from 111 unique localities; details in Suppl. material 1.

##### Diagnosis.

Like *Amastigogonus elephas* sp. n. in having a telopodite with a subquadrate extension of the posterobasal margin and a posteriorly curving pseudoflagellum; distinguished from *Amastigogonus elephas* sp. n. in the pseudoflagellum lacking a small, tooth-like, distally directed extension on the tip.

##### Description.

Mature males observed with (38+4) rings, midbody diameter 2.2 mm to (55+1) rings, 3.2 mm in single 1-month pitfall sample, QVM 23:54197. Cardo not deeper posteriorly. Leg 7 (only) with elongated coxa. Prefemoral pad ca 3/4 femur length. Striae on posterior metazonites reaching ca 3/4 of ozopore height.

Coxite process on anterior gonopod (Fig. 8D) with small portion of posterodistal margin extended as rounded tab, sharply folded over laterally. Telopodite with posterobasal surface produced posteriorly as subquadrate flange (Fig. 8D; fl) and with single row of prominent setae on posterior side of medial thickening, on anterior side of thickening continued to telopodite apex as short row of more closely spaced setae (not shown in Fig. 8D). Pseudoflagellum arising at ca 2/3 telopodite height, ca 1/2 width of telopodite at base, curving posterodistally, the apex narrowing to truncate, posterobasally directed tip.

##### Distribution.

Widespread in forested and some non-forested habitats in western and southern Tasmania and on the Central Plateau (Fig. 7A) from sea level to at least 1260 m; also found on Hunter Island in the Hunter Group in western Bass Strait. Overlaps (with some parapatry?) with *Amastigogonus hellyeri* sp. n. in northwest Tasmania, and with *Amastigogonus fossuliger* on the Central Plateau and possibly in far southern Tasmania (see distribution notes for *Amastigogonus fossuliger*).

##### Remarks.

Gervais (1847) described *Iulus Verreauxii* from material in the Muséum national d’Histoire naturelle in Paris. A presumed holotype was still in the Muséum more than 150 years later and was redescribed and illustrated by Mauriès, Golovatch and Hoffman (2001), who assigned the species to *Amastigogonus*. The type material had probably been collected on Mt Wellington by the naturalist Jules Pierre Verreaux ca 1843 during his residency in Tasmania (Maiden 1910: 153).

Mauriès, Golovatch and Hoffman (2001: 585) refer to a “very faint axial line” on the promentum of the *Amastigogonus verreauxii* holotype. The line appears to be an artefact of long preservation, as I have not observed it in any *Amastigogonus verreauxii* specimens.

*Amastigogonus verreauxii* varies remarkably little in size or gonopod details over its large range.

#### Atelomastix

Taxon classificationAnimaliaSpirostreptidaIulomorphidae

Attems, 1911

Atelomastix Attems 1911: 183 (in genus key), 192 (first description); 1926: 206; 1928: 312. Verhoeff 1913: 59; 1924: 74, 83; 1932: 1728, 1732, 1735, 1741; 1944: 33. Jeekel 1971: 107 (type species designated); 1985: 106; 2009: 31. Hoffman 1980: 91. Mauriès 1987: 196, 198. Korsós and Johns 2009: 3. Edward and Harvey 2010: 6. Korsós and Read 2012: 44.

##### Type species.

*Atelomastix albanyensis* Attems, 1911, by subsequent designation.

##### Other assigned species.

*Atelomastix albanyensis* Attems, 1911, *Atelomastix anancita* Edward & Harvey, 2010, *Atelomastix attemsi* Edward & Harvey, 2010, *Atelomastix bamfordi* Edward & Harvey, 2010, *Atelomastix bonhami* sp. n., *Atelomastix brennani* Edward & Harvey, 2010, *Atelomastix culleni* Edward & Harvey, 2010, *Atelomastix danksi* Edward & Harvey, 2010, *Atelomastix dendritica* Edward & Harvey, 2010, *Atelomastix ellenae* Edward & Harvey, 2010, *Atelomastix flavognatha* Edward & Harvey, 2010, *Atelomastix francesae* Edward & Harvey, 2010, *Atelomastix gibsoni* Edward & Harvey, 2010, *Atelomastix grandis* Edward & Harvey, 2010, *Atelomastix julianneae* Edward & Harvey, 2010, *Atelomastix lengae* Edward & Harvey, 2010, *Atelomastix longbottomi* Edward & Harvey, 2010, *Atelomastix mainae* Edward & Harvey, 2010, *Atelomastix melindae* Edward & Harvey, 2010, *Atelomastix montana* Edward & Harvey, 2010, *Atelomastix nigrescens* Attems, 1911, *Atelomastix poustiei* Edward & Harvey, 2010, *Atelomastix priona* Edward & Harvey, 2010, *Atelomastix psittacina* Edward & Harvey, 2010, *Atelomastix rubricephala* Edward & Harvey, 2010, *Atelomastix sarahae* Edward & Harvey, 2010, *Atelomastix smithi* sp. n., *Atelomastix solitaria* Jeekel, 2009, *Atelomastix tigrina* Edward & Harvey, 2010, *Atelomastix tumula* Edward & Harvey, 2010.

#### Atelomastix bonhami sp. n.

Taxon classificationAnimaliaSpirostreptidaIulomorphidae

http://zoobank.org/BD14A17E-4688-4E7E-AA1D-CF02AC0612F3

Figs 9 , 10A, C, D

##### Holotype.

Male, White Spur, Tas, -42.7764 146.0369 ±100 m [label “DN 211 634” (= 55G 421100 5263400, AGD66)], 320 m a.s.l., 2 February 1994, R. Mesibov, QVM 23:54460.

##### Paratypes.

1 male, 1 female, details as for holotype, QVM 23:54176.

##### Other material.

7 males, 5 females and 5 juveniles from 7 unique localities in Tasmania; details in Suppl. material 1.

##### Diagnosis.

Most similar to *Atelomastix gibsoni* Edward & Harvey, 2010 from the Ravensthorpe Ranges in Western Australia; both species have a wedge-shaped sclerite “b” and a non-bifurcate sclerite “c”. Differences between *Atelomastix bonhami*/*Atelomastix gibsoni*: rounded tab present under sclerite “a” near pseudoflagellum/no tab; anterior corner of distal margin of sclerite “b” higher than posterior corner/anterior corner lower than posterior corner; sclerite “c” taller than sclerite “b”/ sclerite “c” shorter than sclerite “b”.

##### Description.

*Atelomastix* as a genus has been well characterised by Edward and Harvey (2010), who described or redescribed 27 species from Western Australia. *Atelomastix bonhami* sp. n. fits the genus description and only key details are noted here.

Colour in alcohol variable, dark blueish grey to dark brown anteriorly on metazonites, pale posteriorly; some specimens largely brown. Ocelli 30-40 in 4-5 horizontal rows. Mature males with (37+5) rings, 2.0 mm midbody diameter to (60+1) rings, 2.3 mm. Longitudinal striae on metazonites meeting suture almost at right angle. Female substantially more robust than male with similar ring number, e.g. male with (51+1) rings, midbody diameter 2.2 mm vs female with (52+1) rings, midbody diameter 2.8 mm, both in QVM 23:54140.

**Figure 9.**
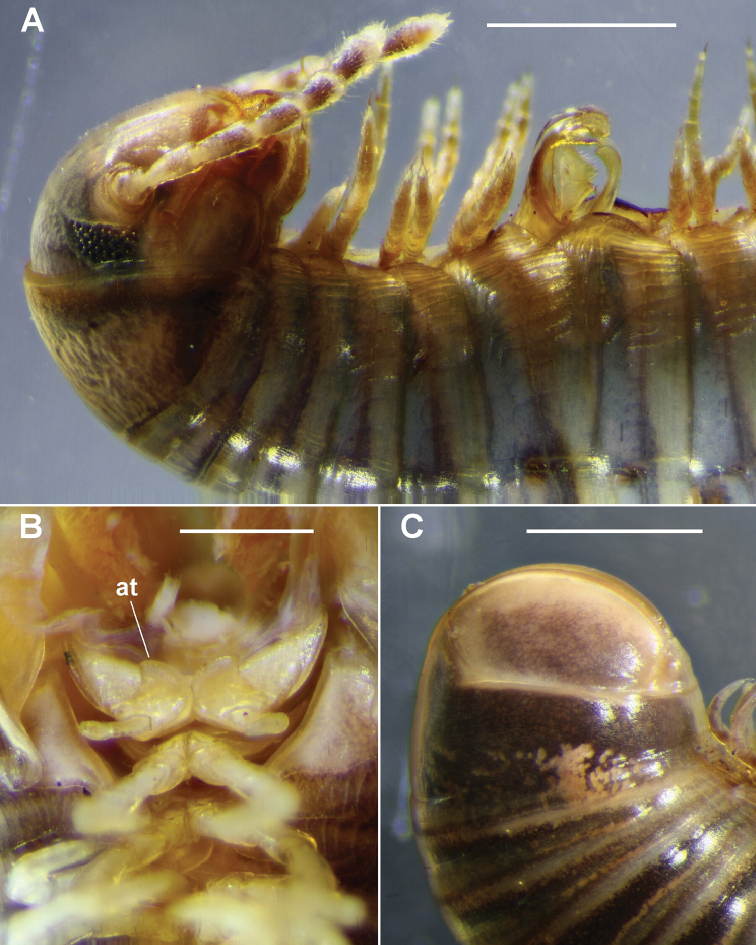
*Atelomastix bonhami* sp. n., males. **A** Left lateral view showing partly everted gonopods; QVM 23:54175 **B** Ventral view of partly dissected specimen showing legpair 1 in situ; at = anterior tab on prefemur; QVM 23:54174 **C** Left lateral view of telson; QVM 23:54140. Scale bars: **A, C** = 1.0 mm, **B** = 0.5 mm.

**Figure 10.**
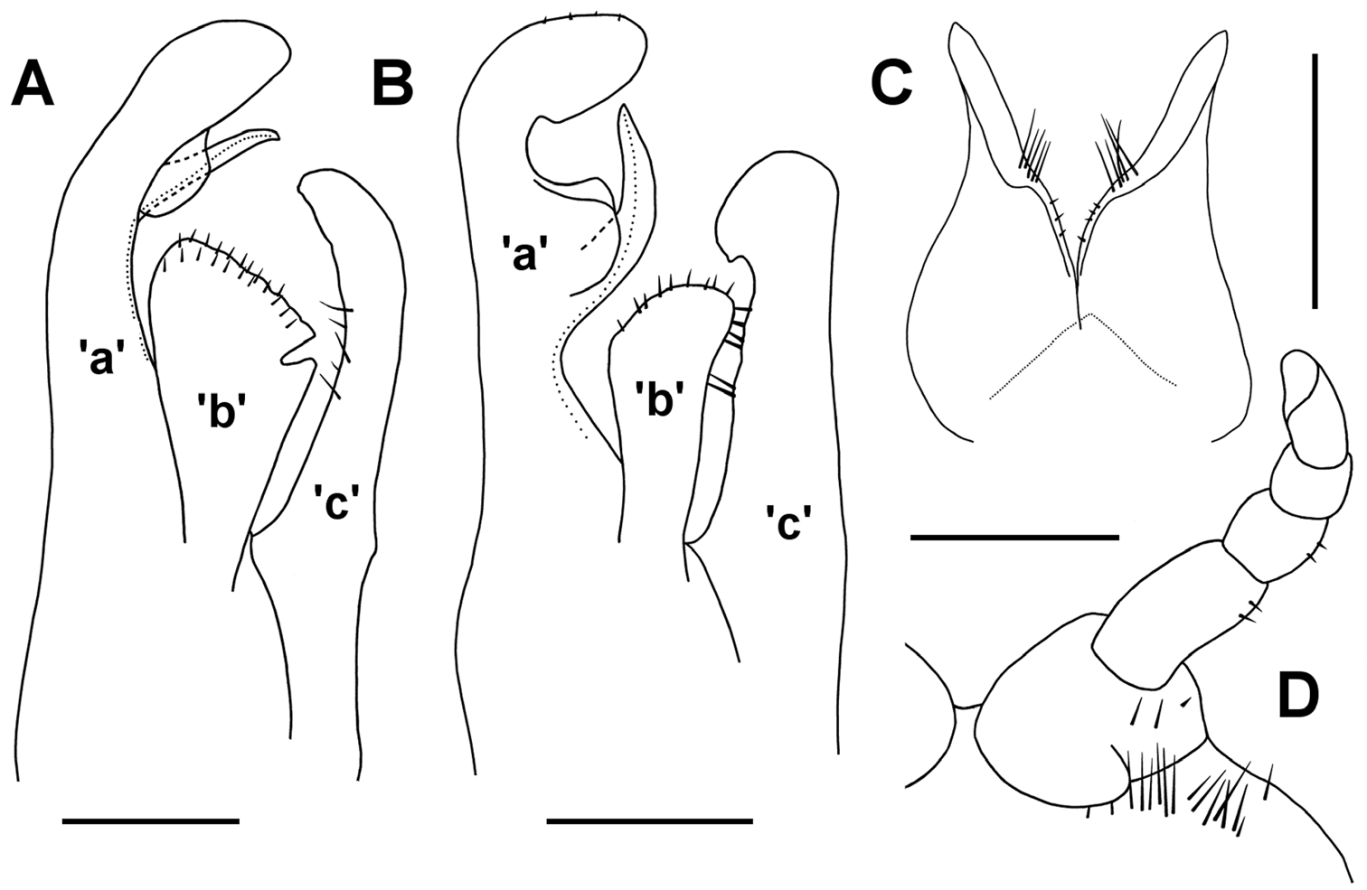
**A, C, D** *Atelomastix bonhami* sp. n., male, QVM 23:54174 **B** *Atelomastix smithi*, sp. n., male paratype, QVM 23:24959 **A, B** Left anterior gonopod, medial view; ps = pseudoflagellum, t = process “a” tab; dotted line indicates prostatic groove **C** Posterior gonopods, posterior view **D** Left leg 1, anterior view; at = anterior tab on prefemur. Scale bars = 0.25 mm.

Male gonopod aperture with sides slightly raised (Fig. 9A). Anterior gonopods nearly touching along midline. Sclerite “a” (Fig. 10A) curving posteriorly with pseudoflagellum (Fig. 10A; ps) arising at ca 3/4 gonopod height, extending posteriorly and slightly distally and gradually tapering to rounded apex; a thin, rounded tab (Fig. 10A; t) arising just distal to pseudoflagellum origin along sclerite midline. Sclerite “b” arising medially at ca 1/3 gonopod height, ca 3x as wide distally as at origin, thickly lamellar with distal half slightly bent medially, the distal margin with rounded anterior corner, sloping posterobasally with small, rounded notch at posterior corner; a double row of small, short setae near distal margin. Sclerite “c” more or less cylindrical basally, the apex curving slightly anteriorly and spatulate, the concave surface facing anterolaterally; a few long setae on anterior surface of sclerite at level of sclerite “b” distal margin.

Posterior gonopods (Fig. 10C) appressed basally along midline, 1/3-1/2 anterior gonopod height. Each posterior gonopod basally conical, tapering and with anterolateral extension; extension slightly flattened with a few long, mediodistally directed setae near base of extension on posteromedial surface and a few short setae at ca 1/2 gonopod height on medial surface.

Leg 1 (Fig. 10D) with prefemur wider than long, slightly tapering, with triangular tab (Figs 9B, 10D; at) on anteromedial surface of prefemur; relative lengths of podomeres femur>prefemur>tarsus>postfemur>tibia; tarsus distally excavate on anteromedial surface; prefemur, femur and postfemur with a few small setae laterally. Legpair 1 clearly separated on coxosternite, the latter with a field of long setae anterolateral to each prefemur.

Posterolateral margin of preanal ring meets epiproct margin at obtuse angle, making anal valves appear more prominent in lateral view than in other Tasmanian Iulomorphidae (Fig. 9C).

##### Distribution.

Known from wet forest, rainforest and scrub over ca 1500 km^2^ in southwest Tasmania at elevations ca 300-1100 m (Fig. 7D).

##### Name.

For Kevin Bonham, Tasmanian malacologist and diligent millipede hunter, who collected this species at two remote sites in 2016; noun in the genitive case.

##### Remarks.

The posterior gonopod of *Atelomastix bonhami* sp. n. is similar to that of *Atelomastix nigrescens* as illustrated in figs 13, 14 and 118 of Edward and Harvey (2010), and leg 1 of *Atelomastix bonhami* sp. n. closely resembles leg 1 of *Atelomastix solitaria* as illustrated in fig. 2 of Jeekel (2009).

Jeekel (2009: figs 3, 4) does not show the *Atelomastix solitaria* anterior gonopod in lateral view, so the shapes of the sclerites are unclear. From Jeekel’s description of *Atelomastix solitaria* and his fig. 4, it appears that sclerite “c” is sharply bent with an acuminate apex, unlike the smoothly curving, apically rounded sclerite “c” of *Atelomastix bonhami* sp. n. and the following species, and is shorter than “b”, rather than longer as in *Atelomastix bonhami* sp. n. and the following species.

#### Atelomastix smithi sp. n.

Taxon classificationAnimaliaSpirostreptidaIulomorphidae

http://zoobank.org/29E9625F-5B3F-4325-BF06-11694A4D9BC2

Fig. 10B

##### Holotype.

Male, Ooze Lake, Tas, -43.5003 146.7019 ±100 m [label “DM 758 834” (= 55G 475800 5183400, AGD66)], 870 m a.s.l., 16 February 1988, S.J. Smith, QVM 23:54179; dissected.

##### Paratypes.

2 males and 1 juvenile, details as for holotype, QVM 23:54484; 1 male, Promontory Lake, Tas, -43.1667 146.3653 ±1 km [label “DN 483 203” (= 55G 448300 5220300, AGD66)], 25 February 2004, P. Sugden, QVM 23:24959.

##### Other material.

None.

##### Diagnosis.

Readily distinguished from the otherwise similar *Atelomastix bonhami* sp. n. by the pseudoflagellum curving distally; this difference can be seen in undissected males.

##### Description.

As for *Atelomastix bonhami* sp. n., but the two known males with 40-50 ocelli in 5-6 horizontal rows; holotype with (54+0) rings, 2.2 mm midbody diameter, paratype with (47+1) rings, 2.1 mm. Females (QVM 23:54484) more robust than males: (42+1) and (47+0) rings, both 2.5 mm in midbody diameter.

Anterior gonopod (Fig. 10B) with sclerite “a” bending posteriorly near bluntly rounded, thickened apex; pseudoflagellum (Fig. 10B; ps) arising at ca 3/4 gonopod height, curving smoothly distally, bending medially and terminating just beneath sclerite “a” apex; a longitudinally divided, thickened tab (Fig. 10B; t) arising just distal to pseudoflagellum origin along sclerite “a” midline. Sclerite “b” arising medially at ca 1/3 gonopod height, ca 2x as wide distally as at origin, thickly lamellar, the distal margin with rounded corners, sloping anterobasally; a double row of small, short setae near distal margin. Sclerite “c” more or less cylindrical, slightly tapered, apex thickened with shallow notch on anterior surface; a few long setae on anterior surface of sclerite at level of sclerite “b” apex.

##### Distribution.

Known from two localities ca 45 km apart at ca 900 m elevation, south and west of the known range of *Atelomastix bonhami* sp. n. in southwest Tasmania (Fig. 7D). *Atelomastix smithi* sp. n. is likely to be more widely distributed in this little-sampled wilderness area.

##### Name.

For Steven J. Smith, first collector of this species and formerly a senior zoologist with the Tasmanian Parks and Wildlife Service; noun in the genitive case.

#### Equestrigonus gen. n.

Taxon classificationAnimaliaSpirostreptidaIulomorphidae

http://zoobank.org/0FB5A7A5-C4DE-4C93-A17A-8D857A7C9B4A

##### Diagnosis.

**Figure 11.**
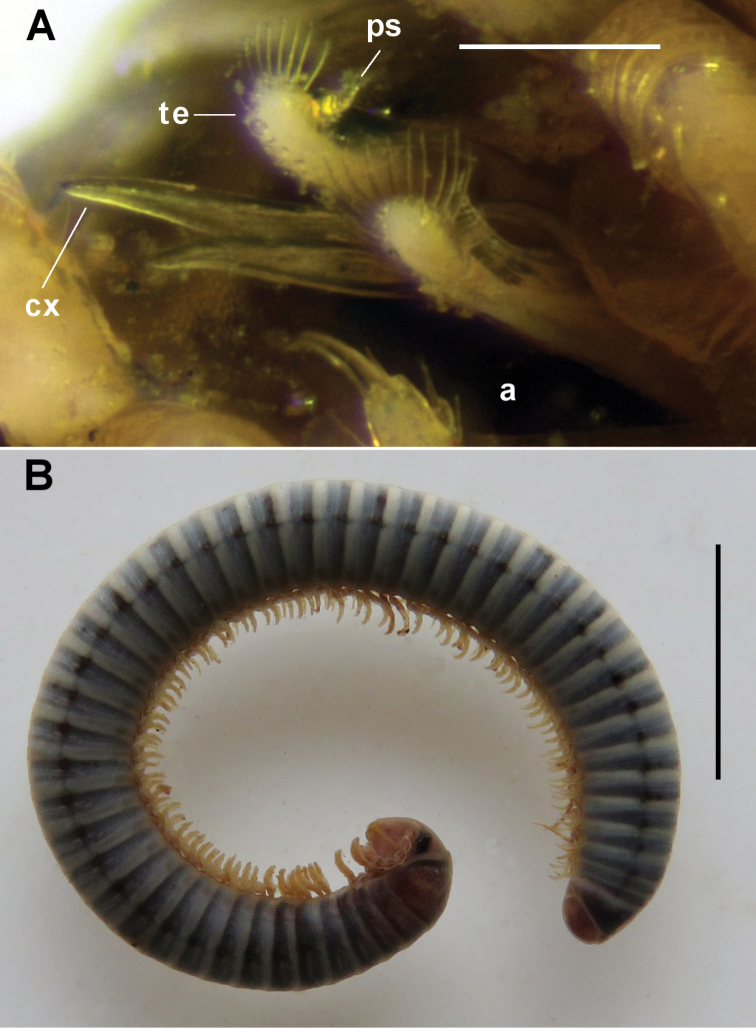
*Equestrigonus tasmaniensis* gen. n., sp. n. **A** Gonopod aperture (a) of male paratype QVM 23:11638, left ventrolateral view, showing protruding tips of anterior gonopods. cx = right coxite tip (“prick spur”), te = right telopodite tip (“rowel spur”), ps = pseudoflagellum
**B** Male holotype QVM 23:54173, habitus. Scale bars: **A** = 0.2 mm, **B** = 5 mm.

**Figure 12.**
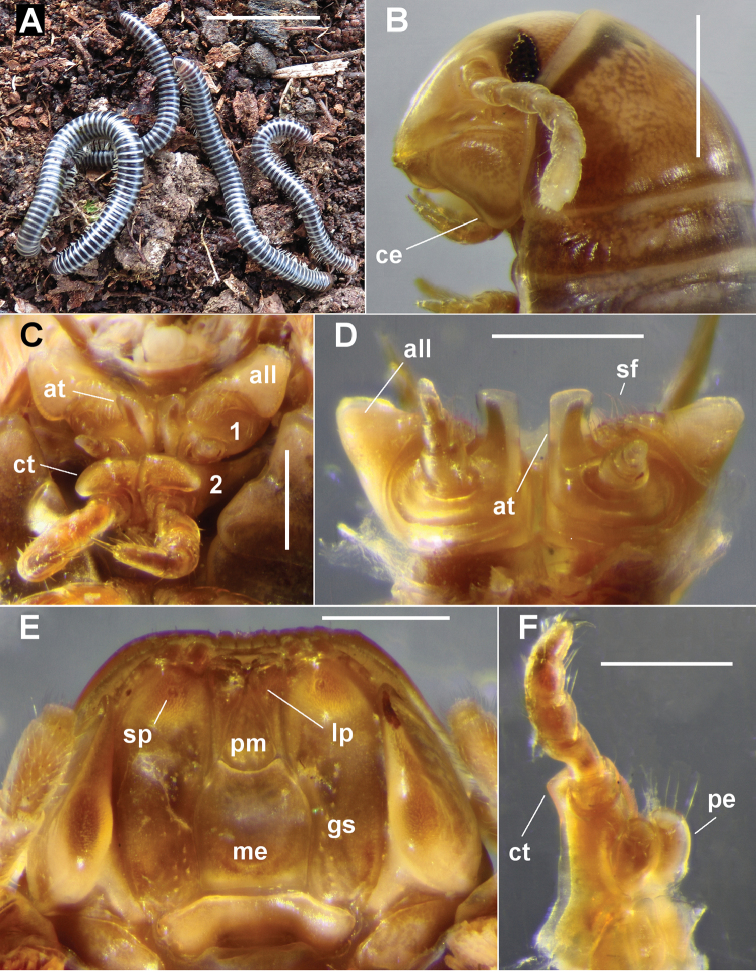
*Equestrigonus tasmaniensis* gen. n., sp. n. **A** Living animals before preservation in QVM 23:54467 **B–F** male paratype ex QVM 23:54094 **B** Head, left lateral view, showing ventral projection (ce) of cardo. **C** Dissection with head removed, ventral view, showing leg 1 complex and legs 2 in situ. all = anterolateral extension of leg 1 coxosternite, at = anterior tab of leg 1 prefemur, ct = coxal tab of leg 2
**D** Dissected leg 1 complex, posteroventral view; sf = field of setae
**E** Gnathochilarium, ventral view; gs = gnathochilarial stipes, lp = lingual plate, me = mentum, pm = promentum, sp = pit on gnathochilarial stipes
**F** Right leg 2, right posterolateral view (left leg removed for clarity); pe = left leg penis. Scale bars: **A** = 10 mm, **B** = 1.0 mm; **C–F** = 0.5 mm.

**Figure 13.**
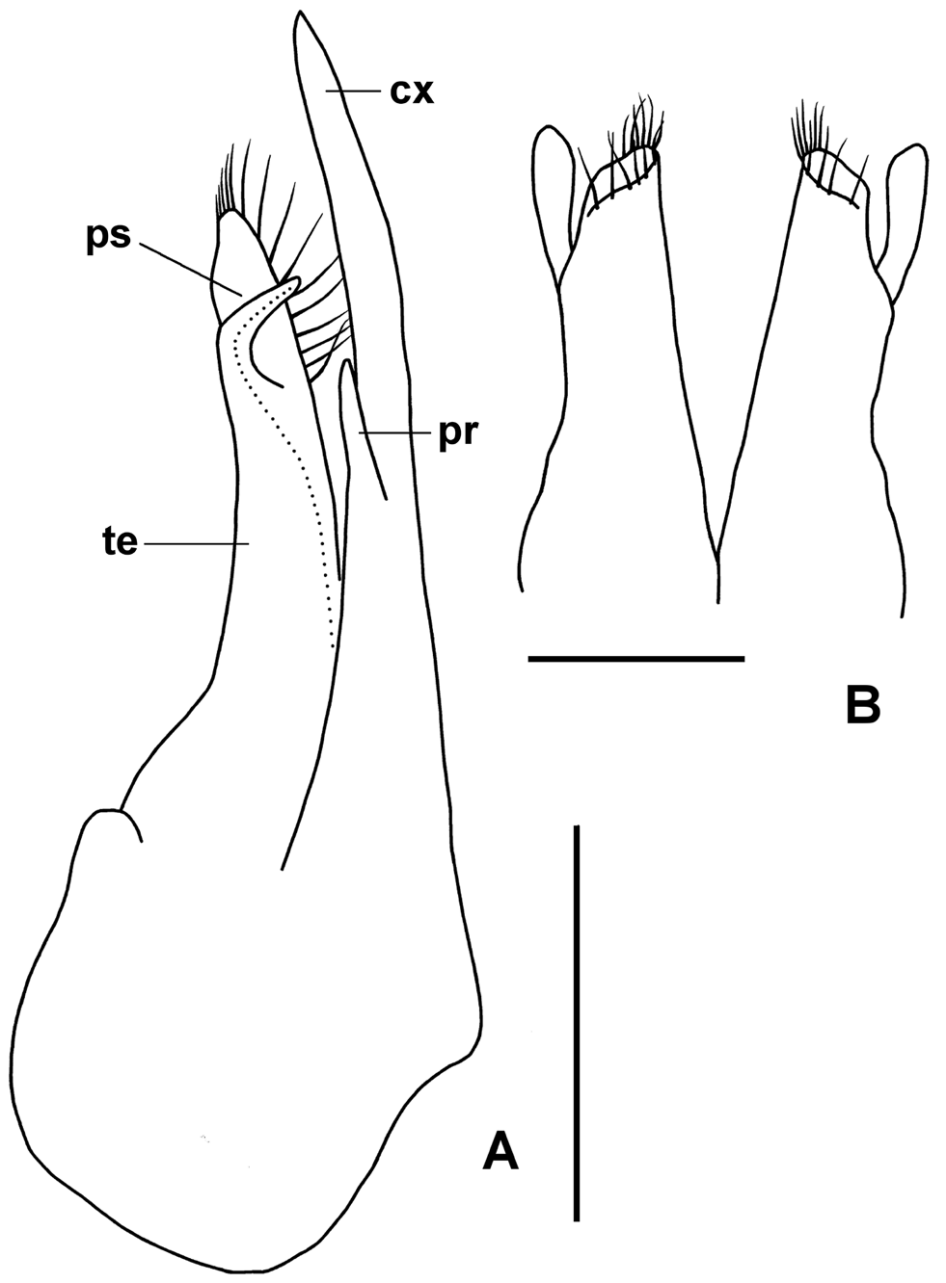
*Equestrigonus tasmaniensis* gen. n., sp. n., QVM 23:54467. **A** Anterior and slightly medial view of left anterior gonopod; cx = coxite, pr = small process on coxite, te = telopodite, dotted line indicates prostatic groove ending on pseudoflagellum (ps) **B** Posterior view of posterior gonopods. Scale bars: **A** = 0.5 mm, **B** = 0.25 mm.

Like *Amastigogonus* and *Victoriocambala* Verhoeff, 1944 in having greatly reduced posterior gonopods with a single lateral process, and relatively simple, two-branched anterior gonopods with the prostatic groove entering a pseudoflagellum on the telopodite. Distinguished from the other two genera by the form of the anterior gonopods (Figs 11A, 13A): coxite process a bluntly pointed rod, longer than telopodite; telopodite slender with a distal, spreading crown of setae. Distinguished from all other Tasmanian Iulomorphidae by the posteroventral extension of the cardo (Fig. 12B; ce) in males.

##### Type species.

*Equestrigonus tasmaniensis* sp. n., by present designation.

##### Name.

From Latin *equestris*, genitive singular of *equester*, pertaining to horse-riding, plus -*gonus*, a suffix commonly used in millipede names, referring to the gonopods; masculine gender. The tips of the anterior gonopod of the type species (Fig. 11A) resemble the ends of prick and rowel spurs.

#### Equestrigonus tasmaniensis sp. n.

Taxon classificationAnimaliaSpirostreptidaIulomorphidae

http://zoobank.org/3C11041C-35F8-415A-99C9-DE4872950642

Figs 11 , 12 , 13

##### Holotype.

Male, Sideling Range, Tas, -41.2358 147.4131 ±100 m [label “EQ 345 348” (= 55G 534500 5434800, AGD66)], 550 m a.s.l., 7 July 1993, T. Kingston, QVM 23:54173 (ex 23:11638).

##### Paratypes.

3 males, Sideling Range, Tas, -41.2303 147.4117 ±100 m [label “EQ 344 354” (= 55G 534400 5435400, AGD66)], 550 m a.s.l., 15 June 1992, T. Kingston and R. D’Orazio, QVM 23:12719; 6 males, same details but 13 September 1993, T. Kingston et al., QVM 23:54057; 6 males, same locality but -41.2411 147.4106 ±100 m [label “EQ 343 342” (= 55G 534300 5434200, AGD66)], 540 m a.s.l., 14 September 1993, T. Kingston et al., QVM 23:54094; 2 males, same locality but -41.2394 147.4083 ±100 m [label “EQ 341 344” (= 55G 534100 5434400, AGD66)], 500 m a.s.l., 14 September 1993, T. Kingston et al., QVM 23:54095; 1 male, details as for holotype, 23:11637; 1 male, details as for holotype, QVM 23:11638.

##### Other material.

1013 males, 82 probable females and 12 possible juveniles from 68 unique localities in Tasmania; details in Suppl. material 1.

##### Description.

Living animals (Fig. 12A) have dark grey rings with annular pale band at rear of each metazonite; head, collum and last 1-2 rings before telson tinged with light brown; legs pale. With long storage in alcohol, ring colour fades to dark blue-grey, annular band darkens to light grey; often with brownish tinge on anterior rings; head and legs pale golden yellow (Fig. 11B).

Mature males (Fig. 11B) with (34+4) rings, 2.0 mm midbody diameter to (50+1) rings, 3.0 mm. Head (Fig. 12B) smooth, frons flattened, vertigial sulcus reaching to level of dorsalmost ocellar row. Posteroventral margin of cardo thickened and extended ventrally as large, rounded lobe (Fig. 12B; ce). Ocellar area narrow-triangular, triangle apex medial; ca 27 ocelli in 4 more or less regular horizontal rows, dorsal>ventral typically 9+8+6+4. Antennae short, barely reaching past posterior edge of collum when manipulated dorsally; relative antennomere lengths (2=3)>6>(4=5); antennomere 6 widest; 4 apical cones; socket ca 1 socket diameter from lateral margin of head capsule. Gnathochilarium (Fig. 12E) with lateral edges of mentum (Fig. 12E, me) slightly convex, mentum wider than combined lingual plates (Fig. 12E, lp); mentum-promentum (Fig. 12E, pm) junction nearly straight or slightly concave anteriorly; a prominent pit (Fig. 12E, sp) with small seta anteriorly on each gnathochilarial stipes (Fig. 12E, gs). Collum convex, laterally narrowing with rounded corner, margins straight. Ventral margin of ring 2 swollen posteriorly. Prozonites only slightly narrower than metazonites; prozonites with weakly defined annular striae anteriorly; suture weakly defined; fine longitudinal striae on lower half of metazonite, anterior end of each stria bent obliquely upwards towards suture; prozonites and metazonites with surface otherwise smooth, free of setae. Ozopores small, beginning ring 6, opening just above 1/2 ring height at ca 1/3 the distance between suture and posterior metazonite margin. Limbus lamellar, undivided. Preanal ring smooth, epiproct broadly rounded, extending slightly over anal valves; hypoproct with margin slightly convex dorsally. Midbody legs short, ca 2/3 ring diameter when extended; relative podomere lengths (prefemur=femur)>tarsus>(postfemur=tibia), claw ca 1/2 tarsus length. Prefemur distally with ventral flattening and conical prefemoral pad ca 1/2 femur length on midbody legs; pads first appear on ring 5 legs, diminish in posterior 1/3 of body and are greatly reduced or absent on last few legpairs.

Legpair 1 (Fig. 12C, D) separate on coxosternite, each leg 1 composed of broad basal and leg-like distal portion. Basal portion here assumed to be prefemur; widest at base, tapering medially, extending anterolaterally at base as large lobe (Fig. 12D; all) with a few distal setae; prefemur marked with several quasi-annular chitinous ridges and anteriorly bearing small field of coarse setae (Fig. 12D; sf), with large subquadrate tab (Fig. 12D; at) extending anteriorly. Distal portion with 3 well-demarcated podomeres, here assumed to be femur, postfemur and fused tibia+tarsus; femur widest, the tibia+tarsus narrowest and longest and with faint annular subdivision; all 3 podomeres sparsely and shortly setose; no claw.

Leg 2 (Fig. 12C, F) incrassate with large claw, prefemur reduced, coxa expanded anteriorly and distally with thick, tab-like apex (Figs 12C, F; ct); penis (Fig. 12F; pe) arising basally on posterior coxal surface, barrel-shaped with a few long setae in distal, marginal crown.

Coxae not elongated on near-aperture legs. Rear portion of gonopod aperture flat, not raised behind gonopods.

Anterior gonopods (Figs 11A, 13A) with coxite process (Figs 11A, 13A; cx) tapering to blunt spine extending further distally than telopodite (Figs 11A, 13A; te), and with short, spine-like process (Fig. 13A; pr) arising on anterior surface at level of pseudoflagellum on telopodite and directed distally. Telopodite tapering strongly, apex spatulate with spreading, marginal crown of ca 15 long, well-spaced setae; pseudoflagellum (Figs 11A, 13A; ps) branching off at ca 3/4 telopodite height on anterior surface, tapering to blunt tip and curving medially to terminate proximal to telopodite apex.

Posterior gonopods (Fig. 13B) ca 1/2 length of anterior gonopods, tapering from base, apex truncate distolaterally with apical crown of long setae; lateral process arising at ca 1/2 gonopod height, terminating at same level as body of gonopod, tip slightly expanded and flattened with convex distal margin.

Mature females (specimens in QVM 23:54050 and 23:54467) a little larger than males with same ring number; cardo not extended ventrally as in male.

##### Distribution.

Wet eucalypt forest and cool temperate rainforest in northern Tasmania (Fig. 7D), from 50 m to at least 900 m elevation; not yet collected south of 41°30’S latitude. Wanders on the forest floor and climbs tree trunks at night; shelters during the day in and under rotting logs and in leaf litter.

##### Name.

For the occurrence of this species in Tasmania; adjective.

##### Remarks.

The distinctive tips of the anterior gonopods invariably protrude from the gonopod aperture (Fig. 11A), allowing males of *Equestrigonus tasmaniensis* gen. n., sp. n. to be recognised without dissection. Western Tasmanian males are generally a little larger in diameter than eastern males.

## Discussion

I am not certain that *Amastigogonus* and *Equestrigonus* gen. n. are endemic to Tasmania, because the iulomorphid fauna of mainland Australia is still very poorly known. The few well-described iulomorphids from the eastern Australian mainland, closest to Tasmania, were named from a small number of specimens mostly held in non-Australian collections: *Apocoptogonus* Jeekel, 2006 (two species from one locality each in New South Wales), *Atelomastix* (one species from one locality, Victoria), *Dimerogonus* Attems, 1903 (one species from one locality, New South Wales), *Merioproscelum* Verhoeff, 1924 (one species from one locality, Queensland), *Proscelomerion* Verhoeff, 1924 (one species from one locality, Queensland), *Thaumaceratopus* Verhoeff, 1924 (two species from one locality, Queensland) and *Victoriocambala* (one species from one locality, one species from six localities, Victoria) (Mesibov 2006–2017).

Like the dalodesmid Polydesmida genus *Tasmaniosoma* Verhoeff, 1936 (Mesibov 2010, 2015), *Amastigogonus* in Tasmania has its highest species diversity on the east coast of the main island, with several small-range endemics there and some mosaic parapatry. Parts of the east coast, and especially the surrounds of the city of Hobart, have unfortunately been degraded by almost 200 years of clearing, grazing and frequent burning. Native millipede populations have been greatly reduced or eliminated as a result, and in some places have been completely replaced by the introduced *Ommatoiulus moreleti* (Lucas, 1860) and other European julids. The iulomorphid most at risk may be the Hobart-area endemic *Amastigogonus hardyi*, which has not yet been found in the city’s larger conservation reserves.

The recognition of two *Atelomastix* species from a high-rainfall district in Tasmania is an interesting result of the present study. Jeekel (2009: 34) wrote that the discovery of *Atelomastix solitaria* was “a remarkable and unexpected extension of the known range of the genus *Atelomastix* from the south-western area of Western Australia to the western edge of Victoria” and considered it possible that the label locality was accidentally in error. It now seems possible that *Atelomastix* species could also occur in high-rainfall parts of eastern Victoria, and perhaps southern New South Wales.

Using a draft version of this paper, Henrik Enghoff (in litt., 26 July 2016) has identified *Amastigogonus tasmanianus* and *Amastigogonus verreauxii* as millipede species that are parasitised by the fungus *Rickia candelabriformi*s Santamaria et al., 2016 and that were referred to as undetermined Iulomorphidae in Santamaria et al. (2016). The relevant records are included in Suppl. material 1.

## Supplementary Material

XML Treatment for Amastigogonus

XML Treatment for Amastigogonus danpicola

XML Treatment for Amastigogonus elephas

XML Treatment for Amastigogonus fossuliger

XML Treatment for Amastigogonus hardyi

XML Treatment for Amastigogonus hellyeri

XML Treatment for Amastigogonus michaelsae

XML Treatment for Amastigogonus orientalis

XML Treatment for Amastigogonus peninsulensis

XML Treatment for Amastigogonus tasmanianus

XML Treatment for Amastigogonus verreauxii

XML Treatment for Atelomastix

XML Treatment for Atelomastix bonhami

XML Treatment for Atelomastix smithi

XML Treatment for Equestrigonus

XML Treatment for Equestrigonus tasmaniensis

## Supplementary materials

Supplementary material 1 Specimen records of Tasmanian Iulomorphidae Data type: Tab Separated Value File (tsv). Explanation note: Specimen records of Tasmanian Iulomorphidae as of 14 July 2016. This dataset is made available under the Open Database License (http://opendatacommons.org/licenses/odbl/1.0/). The Open Database License (ODbL) is a license agreement intended to allow users to freely share, modify, and use this Dataset while maintaining this same freedom for others, provided that the original source and author(s) are credited.Robert Mesibov
